# Adipose retinol saturase is regulated by β-adrenergic signaling and its deletion impairs lipolysis in adipocytes and acute cold tolerance in mice

**DOI:** 10.1016/j.molmet.2023.101855

**Published:** 2023-12-19

**Authors:** Chen Li, Marie F. Kiefer, Sarah Dittrich, Roberto E. Flores, Yueming Meng, Na Yang, Sascha Wulff, Sabrina Gohlke, Manuela Sommerfeld, Sylvia J. Wowro, Konstantin M. Petricek, Dominic Dürbeck, Leonard Spranger, Knut Mai, Holger Scholz, Tim J. Schulz, Michael Schupp

**Affiliations:** 1 Institute of Pharmacology, Max Rubner Center (MRC) for Cardiovascular Metabolic Renal Research, Charité-Universitätsmedizin Berlin, Corporate Member of Freie Universität Berlin, Humboldt-Universität zu Berlin, Berlin, Germany; 2 Department of Adipocyte Development and Nutrition, German Institute of Human Nutrition, Nuthetal, Germany; 3 Department of Endocrinology and Metabolism, Max Rubner Center (MRC) for Cardiovascular Metabolic Renal Research, Charité-Universitätsmedizin Berlin, Corporate Member of Freie Universität Berlin, Humboldt-Universität zu Berlin, And Berlin Institute of Health, Berlin, Germany; 4 DZHK (German Centre for Cardiovascular Research), Partner Site Berlin, Germany; 5 Institute of Translational Physiology, Charité-Universitätsmedizin Berlin, Corporate Member of Freie Universität Berlin, Humboldt-Universität zu Berlin, Berlin, Germany; 6 German Center for Diabetes Research (DZD), München-Neuherberg, Germany; 7 University of Potsdam, Institute of Nutritional Science, Nuthetal, Germany

**Keywords:** Adipose tissue, Thermogenesis, Retinol saturase, β-adrenergic signaling, Lipolysis, Mitochondria

## Abstract

**Objective:**

Retinol saturase (RetSat) is an endoplasmic reticulum-localized oxidoreductase highly expressed in organs involved in lipid metabolism such as white (WAT) and brown adipose tissue (BAT). Cold exposure was shown to increase RETSAT protein in BAT but its relevance for non-shivering thermogenesis, a process with beneficial effects on metabolic health, is unknown.

**Methods:**

We analyzed the regulation of RetSat expression in white and brown adipocytes and different murine adipose tissue depots upon β-adrenergic stimulation and cold exposure. RetSat function during the differentiation and β-adrenergic stimulation of brown adipocytes was dissected by loss-of-function experiments. Mice with BAT-specific deletion of RetSat were generated and exposed to cold. Gene expression in human WAT was analyzed and the effect of RetSat depletion on adipocyte lipolysis investigated.

**Results:**

We show that cold exposure induces RetSat expression in both WAT and BAT of mice via β-adrenergic signaling. In brown adipocytes, RetSat has minor effects on differentiation but is required for maximal thermogenic gene and protein expression upon β-adrenergic stimulation and mitochondrial respiration. In mice, BAT-specific deletion of RetSat impaired acute but not long-term adaptation to cold exposure. RetSat expression in subcutaneous WAT of humans correlates with the expression of genes related to mitochondrial function. Mechanistically, we found that RetSat depletion impaired β-agonist-induced lipolysis, a major regulator of thermogenic gene expression in adipocytes.

**Conclusions:**

Thus, RetSat expression is under β-adrenergic control and determines thermogenic capacity of brown adipocytes and acute cold tolerance in mice. Modulating RetSat activity may allow for therapeutic interventions towards pathologies with inadequate metabolic activity.

## Highlights

• Adipose RetSat expression is induced by β-adrenergic signaling and cold exposure.
• Loss of RetSat in brown adipocytes reduces thermogenic gene expression.
• Mice lacking RetSat in BAT have impaired acute cold tolerance.
• RetSat depletion in brown and white adipocytes interferes with lipolysis.

## Abbreviations

Abhd5: α/β hydrolase domain containing 5
Adrb3: adrenoreceptor beta 3
Atgl: adipose triglyceride lipase
BAT: brown adipose tissue
β3A: β3 adrenergic receptor agonist
βA: β adrenergic receptor agonist
Cebpb: CCAAT-enhancer-binding protein β
ChREBP: carbohydrate response element binding protein
Cidea: cell death inducing DFFA like effector A
Cox7a1: cytochrome c oxidase subunit 7a1
DCF: 2′,7′-dichloro-dihydrofluorescein-diacetate
Elovl3: elongation of very long chain fatty acids protein 3
ER: endoplasmic reticulum
Fabp4: fatty acid binding protein 4
FDR: false discovery rate
GO: Gene Ontology
H2O2: hydrogen peroxide
HFD: high-fat diet
HSP: heat shock protein
iBAT: interscapular brown adipose tissue
ingWAT: inguinal white adipose tissue
KEGG: Kyoto Encyclopedia of Genes and Genomes
NC: normal chow
NEFA: non-esterified fatty acids
OCR: oxygen consumption rate
pgWAT: perigonadal white adipose tissue
Pparg2: peroxisome proliferator-activated receptor gamma 2
PPARγ: peroxisome proliferator-activated receptor γ
PPRE: PPAR response element
Prdm16: PR domain containing 16
RA: retinoic acid
RAR: retinoic acid receptors
RetSat: Retinol Saturase
RNAseq: RNA sequencing
ROS: reactive oxygen species
subqWAT: subcutaneous white adipose tissue
tert-BH: tert-butyl hydroperoxide
TZD: thiazolidinedione
Ucp1: uncoupling protein 1
UPR: unfolded protein response
WAT: white adipose tissue

## 1 Introduction

Retinol saturase (RetSat) is an endoplasmic reticulum (ER) membrane-associated oxidoreductase that can reduce retinol to 13,14-dihydroretinol [1,2] in a stereospecific manner [3]. RetSat contains an N-terminal signal peptide for ER localization and a dinucleotide binding motif for NAD(P)/FAD, which is essential for its enzymatic activity [2]. RetSat expression is detectable in many tissues and cell types, but shows particularly high expression in metabolically active organs like liver, adipose tissue, intestine, and kidney. In humans, the tissue with highest RetSat expression is adipose tissue and studies in mice showed that it localizes predominantly to the adipocyte fraction of this tissue [2].

During differentiation of white adipocytes, RetSat expression is robustly induced, mediated by the master regulator of adipogenesis, peroxisome proliferator-activated receptor γ (PPARγ) [4] via a PPAR response element (PPRE) within the first intron of the *RetSat* gene [2]. Consistently, PPARγ activation by synthetic thiazolidinedione (TZD) agonists or its depletion by siRNA increases or decreases RetSat expression in adipocytes, respectively, rendering RetSat a canonical PPARγ target gene [2]. Notably, ample RetSat expression in liver and its induction during fasting is driven by the hepatic isoform PPARα [5].

Functionally, RetSat enhances white adipocyte differentiation *in vitro* since its depletion in precursor cells impaired their adipocytic conversion. Accordingly, overexpression of wildtype-, but not of enzymatically inactive RetSat mutants, increased differentiation, suggesting that its oxidoreductase activity is required for promoting adipogenesis [2]. RetSat overexpression associated with increased activity of PPARγ during differentiation, which may be due to the finding that RetSat functions as upstream activator of the lipogenic transcription factor and glucose sensor carbohydrate response element binding protein (ChREBP) [6]. Indeed, ChREBP-driven *de novo* lipogenesis can activate PPARγ and enhance adipocyte differentiation, presumably via increased synthesis of PPARγ-activating lipids [7]. Supplementing its putative product, 13,14-dihydroretinol, to RetSat depleted cells surprisingly failed to rescue adipocyte differentiation or reduced expression of ChREBP target genes in primary hepatocytes, suggesting that RetSat fulfills other enzymatic reactions than dihydroretinol synthesis in these cell types [2,6]. Nevertheless, RetSat may consume cellular retinol, thereby lowering substrate availability for retinoic acid (RA) synthesis or increase dihydroretinoid levels, which are less potent activators of retinoic acid receptors (RAR) than their 13,14 non-saturated retinoid counterparts [8]. Retinol depletion by RetSat was proposed to mediate its pro-ferroptotic activity in cancer cells [9]. In fact, RetSat confers sensitivity to peroxide (H_2_O_2_) exposure in many cell types and its loss-of-function enhances cell survival of peroxide-challenged cells by lowering reactive oxygen species (ROS) production [[10], [11], [12]]. Besides retinol, no other substrates for mammalian RetSat have been identified [13]. However, zebra finch RetSat can reduce a double bond of an apocarotene derivative to produce dihydrogalloxanthin, allowing for spectral fine tuning of the avian retina [14].

In contrast to its role in adipocyte differentiation, mice that lack RetSat show increased body weight and adiposity [12,15], providing evidence that although relevant for adipocyte differentiation *in vitro*, RetSat is not required for *in vivo* adipogenesis. Since body weight and fat mass are determined by many factors, the contribution of adipocyte-expressed RetSat to this phenotype is currently unknown. However, obesity in humans and mice, irrespective of diet-induced or genetic, associates with reduced RetSat expression in white adipose tissue (WAT), implying additional functions in adipose tissue [2] and underlining the earlier reported highly dynamic expression of RetSat in conditions related to type 2 diabetes and insulin resistance as the top differentially-regulated gene [16].

Lipolysis mobilizes triglyceride stores in adipocytes for the release of fatty acids and glycerol [17]. Whereas white adipocytes increase triglyceride hydrolysis especially during fasting, brown adipocytes generate fatty acids primarily upon cold exposure to activate [18] and, depending on the nutritional status [19,20], fuel non-shivering thermogenesis [21,22]. Moreover, lipolysis has been recognized to produce metabolic signals with profound effects on gene expression and function of adipocytes, reaching far beyond the sole generation of substrates for energy or heat production [17,21,23].

A previous study found RETSAT protein abundance in brown adipose tissue (BAT) robustly induced by switching thermoneutrality-acclimated mice at 29 °C–5 °C [24], suggesting relevance of the oxidoreductase for cold adaptation. Specific functions of RetSat in thermogenesis have not been described. We therefore investigated the regulation and function of RetSat in white and brown adipocytes and implicate RetSat in the expression of thermogenic genes and mitochondrial activity. BAT-specific deletion of RetSat in mice impaired acute but not long-term adaptation to cold exposure. In humans, *RetSat* mRNA in subcutaneous (subq) WAT correlates with the expression of genes related to mitochondrial function. We found that RetSat depletion impaired β-adrenergic agonist (βA)-induced lipolysis, which was identified previously as major regulator of thermogenic gene expression in adipocytes [21,23,[25], [26], [27]]. We conclude that RetSat regulates the thermogenic capacity of adipocytes and may allow for novel interventions towards pathologies with inadequate metabolic activity.

## 2 Material and methods

### 2.1 Culturing, differentiation, and treatment of precursor cell lines

Murine iBACs [28] and 3T3-L1 cells [29] were cultured and differentiated as previously described [30]. Suboptimal differentiation of iBACs for evaluating the effects depletion or overexpression of RetSat was induced by the standard hormone cocktail lacking the pharmacological PPARγ activator pioglitazone. Gene silencing was performed by electroporation (Nucleofactor, Lonza) of siRNA oligonucleotides (Eurogentec) (Supplemental Table S1) as before [31], after detaching precursor cells with trypsin or adipocytes with 4× trypsin plus 0.5 mg/ml collagenase P (Roche). Retroviral overexpression of RetSat in precursor cells and Oil Red O staining of adipocytes were carried out as described elsewhere [32]. Cells were incubated with isoproterenol at 10 μM or 1 mM of dibutyryl-cAMP as indicated, supplementing control cells with the respective vehicle solvents. ROS were visualized after loading adipocytes for 20 min with 20 μM of 2′,7′-dichlorodihydrofluorescein-diacetate (DCF) in DMEM containing 2% of fetal bovine serum and with or without a subsequent 40 min exposure to 200 μM of *tert*-butyl hydroperoxide (tert-BH) in serum-free DMEM. ROS fluorescence intensity was analyzed by ImageJ [33]. Glycerol and NEFA concentrations in serum and cell supernatant were determined by commercially available kits (free glycerol reagent Sigma F6428 and NEFA-HR (2) Assay, Wako). Glycerol and NEFA release of cells was normalized to cell number.

### 2.2 Isolation, culturing, differentiation, and treatment of primary brown preadipocytes

Interscapular BAT of newborn mice was excised, minced, and digested with 2 mg/ml collagenase II (Worthington Industries) for 30 min and filtered through a 250 μM nylon mesh. After centrifugation at 300*g* for 10 min, the pelleted stromal vascular fraction was resuspended and seeded in cell culture plates for expansion and subsequent differentiation by using the standard hormone cocktail, supplemented with 5 μM of pioglitazone. Brown adipocytes derived from mice with floxed *RetSat* alleles were infected with adenoviruses expressing Cre-recombinase or GFP [2] using 0.5 μg/ml polylysine in DMEM supplemented with 0.5% bovine serum albumin overnight [34].

### 2.3 Mitochondrial oxygen consumption

RetSat loss-of-function in primary brown adipocytes was induced via adenoviral Cre expression. Adipocytes were analyzed 96 h after inducing RetSat deletion according to the standard Seahorse XF analyzer protocol. Oxygen consumption was normalized to DAPI fluorescence of cellular DNA [35].

### 2.4 Mouse model and characterization

Animal procedures were in accordance with institutional guidelines and approved by the corresponding authorities of the LAGeSo Berlin, protocol number G 0130/17. All lines were backcrossed to C57BL/6J and kept on a standard 12 h light/dark rhythm. Generation of mice with floxed *RetSat* alleles is described elsewhere [36]. Mice were crossed with mice expressing Cre recombinase under the control of the *Ucp1* promoter [37], genotyping primers of genomic DNA isolated from ear biopsies are listed in Supplemental Table S2. Mice were fed normal chow (ssniff R/M-H, V1534-300, Soest, Germany). Obesity was induced by feeding D12492 with 60% kcal/fat (4 IU/g vitamin A, Research Diets, USA). For comparing obese mice to normal chow-fed mice, D12450B with 10% kcal/fat (4 IU/g vitamin A, Research Diets, USA) was used. Single-housed mice were exposed to 4 °C for indicated times in a climate chamber and core body temperature was monitored by a rectal probe. Adipose tissue activation was induced by injecting mice intraperitoneally with 1 mg/kg CL316,243 (Sigma) for 10 consecutive days. Body composition was assessed by NMR (Bruker Minispec Live Mice Analyzer LF50). Intraperitoneal glucose and insulin tolerance tests were performed as described previously [6,38]. Blood glucose was determined by commercial test stripes and a blood drop derived from the tail vein (Contour, Ascensia Diabetes Care). Mice were sacrificed and serum and organs kept for further analyses, adipose tissue depots were excised and weighted.

### 2.5 Isolation and analysis of mRNA expression by quantitative PCR (qPCR) and RNA profiling

Total RNA of mouse tissues or cells was isolated by a standard spin column kit (PeqGOLD total RNA kit, VWR). cDNA was synthesized by using M-MLV (Promega). qPCRs were performed using Sybrgreen PCR Mastermix (Eurogentec) and evaluated by using standard curves. All mRNA expression data were normalized to the expression of murine *Rplp0* or *18s*. qPCR primer sequences are listed in Supplemental Table S2. RNA sequencing (RNAseq) and data processing were carried out by the Berlin Institute of Health genomics core facility, Charité Berlin. In short, interscapular BAT total RNA was DNAse digested and concentrated using the Clean & Concentrator kit (Zymo Research). RNA integrity was evaluated by the RNA ScreenTape system (Agilent). Poly(A) selection was applied to capture mRNA for further processing. Library preparation was carried out with the NEBNext Ultra II RNA library prep kit and module (New England Biolabs). Sequencing was done on a NovaSeq 6000 system by a paired-end approach (2 × 51 cycles). Differentially expressed genes with Ensembl annotation, gene symbol, log2 FC, P-value, and false discovery rate (FDR) were identified using the DESeq2 package and corrected for multiple testing using the Benjamini-Hochberg procedure using default parameters. KEGG pathway and gene ontology enrichment was analyzed by DAVID [39].

### 2.6 Protein isolation and analysis

Whole tissue or cell proteins were isolated and homogenized by standard methods and analyzed by SDS-PAGE as described previously [40]. Protein concentrations were determined by the BCA method (Thermo Scientific). After incubation of membranes with primary antibodies, secondary horseradish-conjugated goat antibodies were added (Supplemental Table S3) and a chemiluminescent substrate kit (Thermo Scientific) used for detection. Densitometric analyses were performed by ImageJ [33].

### 2.7 Tissue histology

Interscapular BAT was fixed with formalin overnight, dehydrated, and embedded in paraffin. Sections of 5 μm were deparaffinized, rehydrated, and stained by hematoxylin and eosin (H&E) using a standard protocol.

### 2.8 BAT lipid peroxidation

Lipid peroxidation in iBAT was determined by the reaction of tissue malondialdehyde (MDA) with thiobarbituric acid for 1 h at 95 °C and the colorimetric analysis of the formed adducts by using a commercially available kit (MAK085, Sigma–Aldrich).

### 2.9 Human adipose tissue biopsies

Patient characteristics, ethical approval, and biopsy sample processing and analysis were described previously [41].

### 2.10 Statistics

Sample sizes in mouse experiments and number of replicates in cell culture experiments were based on previous experience with similar studies but not predetermined. Animals were randomized into vehicle or treatment groups. During mouse characterization, the operator was blinded regarding the mouse genotype. Reported replicates for *in vitro* cell culture experiments are technical replicates. Statistical significance was evaluated with two-tailed unpaired t-tests for two group comparison or with ANOVA for data with more than two groups. Post hoc Sidak and Dunnet tests were performed for multiple-group comparison if ANOVA reached statistical significance as appropriate and *P* < 0.05 was deemed significant. Data are expressed as mean ± SEM. Data analysis was not masked. Outlier calculation was performed for gene expression in mouse tissues using the Grubbs' test using GraphPad Prism 7 (Dotmatics).

## 3 Results

### 3.1 RetSat is highly expressed in brown adipose tissue and induced by cold exposure, β-adrenergic signaling, and feeding high-fat diet

**Figure 1:**
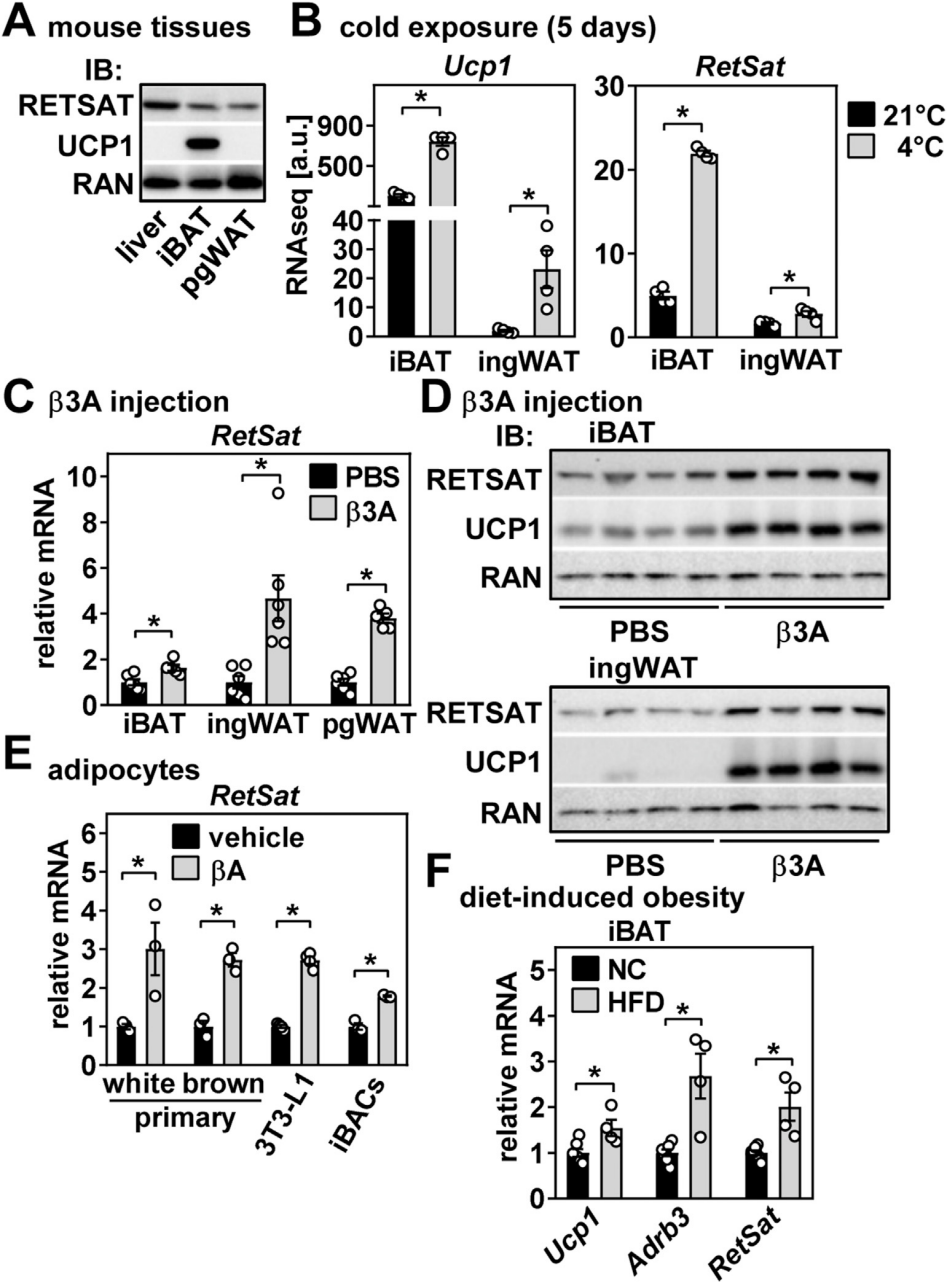
**RetSat is highly expressed in brown adipose tissue and induced by cold exposure, β-adrenergic stimulation, and feeding high-fat diet in mice.** A) Expression of RETSAT and UCP1 in murine liver, interscapular brown adipose tissue (iBAT), and perigonadal white adipose tissue (pgWAT) was analyzed by immunoblotting. RAN protein served as loading control. B) Male mice were exposed to 21 or 4 °C for 5 days and mRNA expression of *Ucp1* and *RetSat* in iBAT and inguinal wgite adipose tissue (ingWAT) determined. C) Male mice were injected with the β3-adrenergic receptor agonist CL-316,243 for 10 consecutive days mRNA expression of *RetSat* and D) protein expression of RETSAT and UCP1 determined. RAN protein served as loading control. E) Differentiated primary white and brown adipocytes were incubated the pan-β-adrenergic receptor agonist isoproterenol for 24 (primary adipocytes, 3T3-L1) or 48 h (immortalized brown adipogenic cells (iBACs)) and mRNA expression of *RetSat* determined by qPCR. F) Expression of indicated genes in iBAT of mice fed for 22 weeks normal chow (NC) or high-fat diet (HFD) was determined by qPCR. In B) (n = 4,4), C) (n = 6,6), E) (all n = 3,3), and F) (n = 7,4), data are presented as individual data points and mean ± sem, ∗*P* < 0.05.

Murine *RetSat* mRNA expression is higher in BAT than in WAT and only surpassed by liver, the organ with highest *RetSat* expression in mice (Supplemental Fig. S1). RETSAT protein abundance was also slightly higher in interscapular (i) BAT than in perigonadal (pg) WAT, but lower than in liver (Figure 1A). Housing mice for 5 days at 4 °C led to the expected increase in uncoupling protein 1 (*Ucp1*) expression in iBAT (Figure 1B, left panel). Similarly, expression of *RetSat* mRNA was markedly induced (Figure 1B, right panel). Hence, we conclude that the previously observed increase in RETSAT protein abundance upon cold exposure [24] is most likely due to increased transcription rather than enhanced RETSAT protein stability. Furthermore, and again mirroring expression of *Ucp1*, we detected cold-inducibility of *RetSat* mRNA also in inguinal (ing) WAT (Figure 1B), implying that the oxidoreductase may be involved in the adaptation of both BAT and WAT depots to cold. We next investigated whether β-adrenergic signaling, known to be pivotal for mediating cold adaptation of adipose tissue [42], may cause upregulation of RetSat. Indeed, daily injections of a specific β_3_-agonist (β3A) for 10 consecutive days induced *RetSat* mRNA and protein in all adipose tissue depots analyzed (Figure 1C,D). This induction appears to be adipocyte-autonomous, since the pan-βA isoproterenol induced *RetSat* mRNA expression in both primary white and brown adipocytes and differentiated 3T3-L1 [29] and differentiated SV40-immortalized brown adipogenic cells (iBACs) [28] (Figure 1E) when stimulated for 24–48 h. Interestingly, short βA incubation periods for 4 h resulted in reduced *RetSat* mRNA expression (Supplemental Fig. S2), indicating exposure time-dependent differences in the regulation of *RetSat* mRNA by βA. Finally, we tested whether diet-induced obesity upon feeding high-fat diet (HFD) regulates *RetSat* expression in iBAT. HFD-fed mice were heavier (50.3 ± 0.6 g vs. 36.7 ± 0.8 g body weight of normal chow (NC)-fed mice, *P* < 0.05) and showed increased expression of *Ucp1* and ‘adrenoreceptor beta 3’ (*Adrb3*) (Figure 1F), consistent with the notion of diet-induced thermogenesis [43,44]. Moreover, we found that expression of *RetSat* in iBAT was higher in obese mice (Figure 1F). Taken together, RetSat is robustly expressed in iBAT and transcriptionally induced by cold and feeding HFD, presumably via β-adrenergic signaling.

### 3.2 RetSat is upregulated during brown adipocyte differentiation and slightly enhances adipogenesis

**Figure 2:**
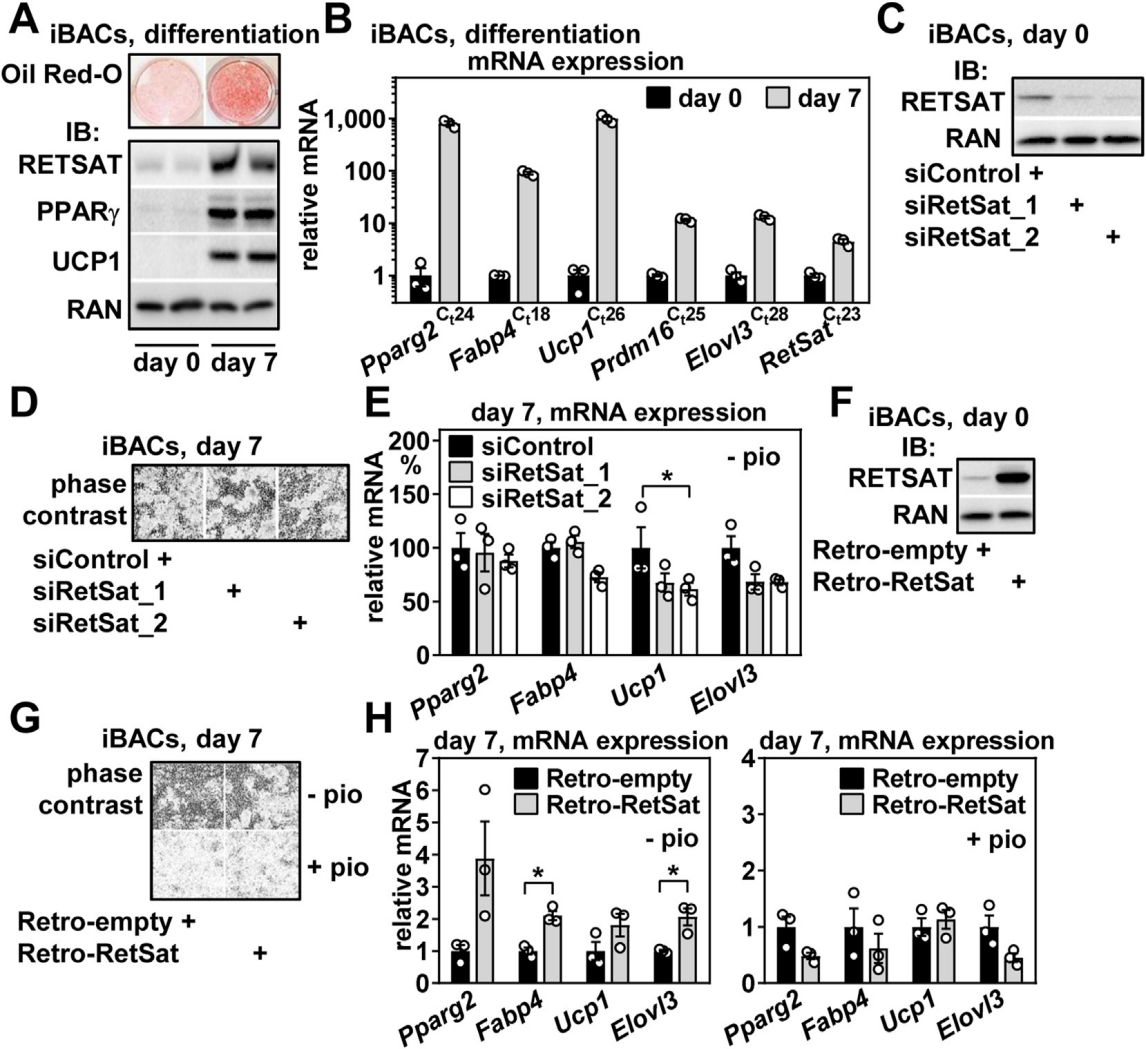
**RetSat is upregulated during brown adipocyte differentiation and slightly enhances adipogenesis.** Immortalized brown adipogenic cells (iBACs) were analyzed for A) intracellular lipids by Oil Red-O staining (top panel) and protein expression of RETSAT, PPARγ, and UCP1 before (day 0) and after (day 7) differentiation (bottom panel). RAN protein served as loading control. B) Cells described in A) were analyzed for mRNA expression of the indicated genes. C_t_ values reflect expression strength in brown adipocytes (lower C_t_ = higher expression). C) Undifferentiated iBACs were electroporated with indicated siRNAs and protein expression of RETSAT analyzed, RAN protein served as loading control. Cells were differentiated and adipocytic conversion assessed by D) phase contrast microscopy and E) mRNA expression of the indicated genes. F) Undifferentiated iBACs were infected with empty or RetSat-encoding retroviruses and protein expression of RETSAT analyzed, RAN protein served as loading control. Cells were differentiated in the absence (-pio) or presence (+pio) of the PPARγ agonist pioglitazone and adipocytic conversion assessed by G) phase contrast microscopy and H) mRNA expression of the indicated genes. In B) (n = 3,3), E) (n = 3,3,3), and H) (n = 3,3), data are presented as individual data points and mean ± sem, ∗*P* < 0.05.

RetSat is upregulated during differentiation of murine 3T3-L1 and human Simpson-Golabi-Behmel syndrome-derived precursor cells [2,15], both adipocytes that show predominantly white characteristics [29,45]. During the differentiation of cultured brown precursor cells, we observed a similar upregulation of RetSat protein (Figure 2A) and mRNA (Figure 2B). Overall expression of RetSat was robust and, extrapolated from gene-specific qPCR C_t_ values and especially in undifferentiated cells, higher than that of more specific adipocyte (*Pparg2*) or brown (*Ucp1*) marker genes (Figure 2A,B). Depletion of RetSat by electroporating two different siRNAs in undifferentiated iBACs (Figure 2C) had little effect on adipocytic conversion, when using a suboptimal differentiation cocktail that lacks the PPARγ activator pioglitazone and that elicits ∼50% differentiation of siControl-treated cells, when assessed by phase contrast microscopy (Figure 2D) and mRNA expression of the differentiation markers *Pparg2* and fatty acid binding protein 4 (*Fabp4*) (Figure 2E). However, both brown marker genes *Ucp1* and ‘elongation of very long chain fatty acids protein 3’ (*Elovl3*) were expressed at lower levels after differentiation of RetSat-depleted cells (Figure 2E). Retroviral RetSat overexpression (Figure 2F), known to promote differentiation of 3T3-L1 precursors cells [2], also led to enhanced differentiation of iBACs (Figure 2G, **top**) and consistently higher expression of differentiation and brown adipocyte marker genes (Figure 2H, left panel). However, increasing the strength of the differentiation cocktail by adding pioglitazone blunted the enhancing effect of overexpressed RetSat on differentiation (Figure 2G, bottom and Figure 2H, right panel), in accordance with findings from differentiating 3T3-L1 cells [2]. Thus, RetSat is induced during the differentiation of brown adipocytes and enhances differentiation, at least in the absence of pharmacological PPARγ activation.

### 3.3 RetSat is required for the maximal induction of thermogenic gene expression and respiration of brown adipocytes

**Figure 3:**
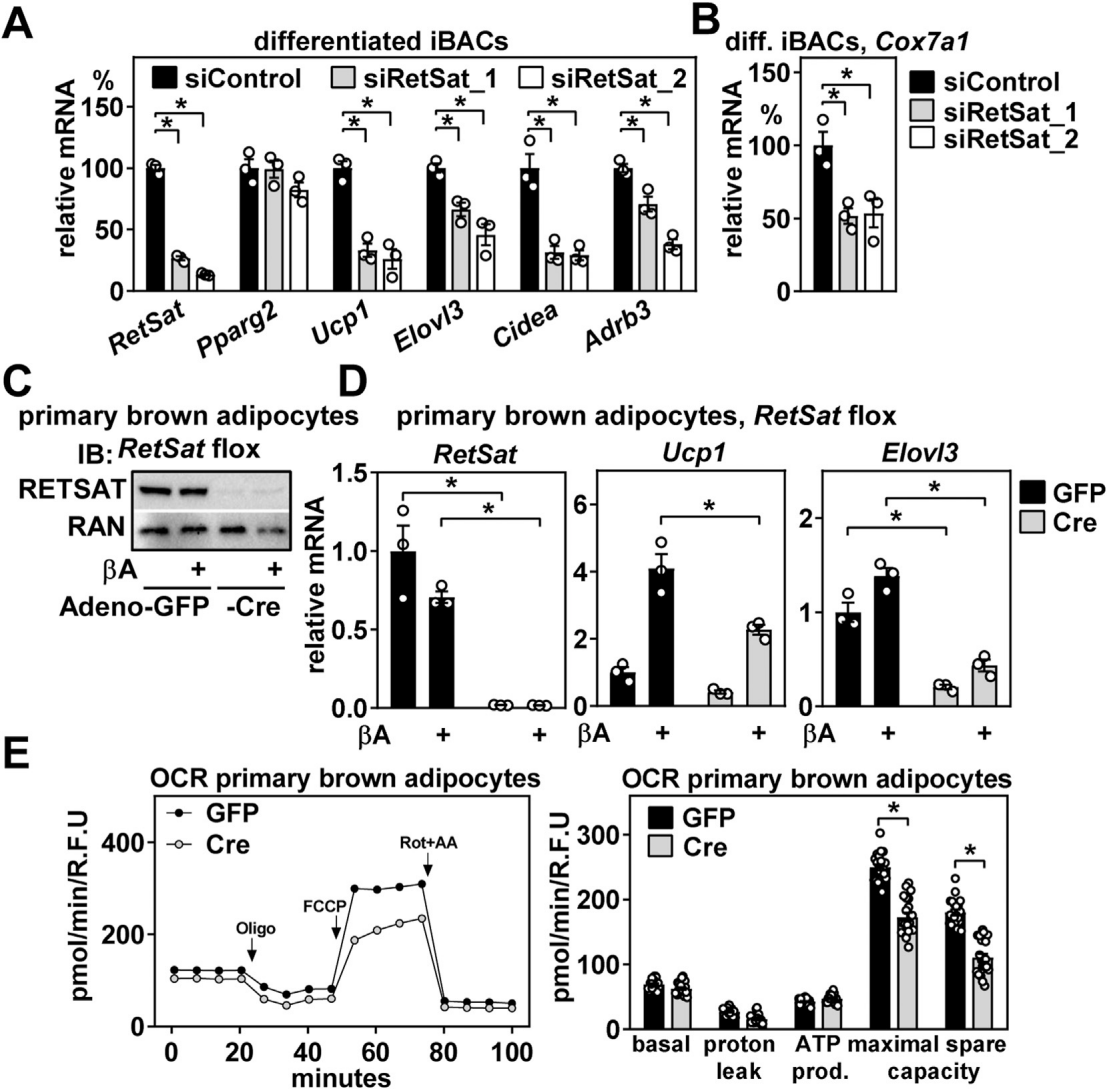
**RetSat is required for the maximal induction of thermogenic gene expression and respiration of brown adipocytes.** A) Differentiated immortalized brown adipogenic cells (iBACs) were electroporated with indicated siRNAs and mRNA expression of *RetSat*, *Pparg2*, and thermogenic genes determined by qPCR. B) *Cox7a1* mRNA expression in iBACs described in A) was analyzed by qPCR. Differentiated primary brown adipocytes with floxed *RetSat* alleles were infected with adenoviruses expressing Cre recombinase or GFP. 4 days later, adipocytes were incubated with 10 μM βA for 4 h and C) protein expression of RETSAT determined by immunobloting, RAN served as loading control. D) mRNA expression of *RetSat*, *Ucp1*, and *Elovl3* was analyzed by qPCR. E) Oxygen consumption rate (OCR) of primary brown adipocytes with or without *RetSat* deletion was analyzed by Seahorse XF Analyzer (left panel) and evaluated stage-specifically (right panel). In A), B), D) (all n = 3,3,3,3), and E) (n = 21,21), data are presented as individual data points and mean ± sem, ∗*P* < 0.05.

Reduced expression of thermogenic genes despite comparable differentiation of RetSat-depleted iBAC precursors (Figure 2E) prompted further analysis of RetSat's function in mature brown adipocytes. RetSat knockdown by the two different siRNAs had little effect on *Pparg2* mRNA expression but downregulated all analyzed genes related to brown adipogenesis and thermogenesis, including ‘cell death inducing DFFA like effector A’ (*Cidea*) and ‘adrenoreceptor beta 3’(*Adrb3*) (Figure 3A). Furthermore, induction of *Ucp1* mRNA expression by a 4-hour treatment with isoproterenol was markedly blunted in RetSat-depleted adipocytes (Supplemental Fig. S3A) and reflected in decreased UCP1 protein levels (Supplemental Fig. S3B and its densitometric analysis in S3C). Lower UCP1 protein expression was sustained after long-term depletion of RetSat, analyzed at 7 days instead of 72 h after the electroporation of siRNA in mature brown adipocytes (Supplemental Fig. S3D), arguing against a short-term and transient effect of RetSat depletion on thermogenic gene expression. Expression of cytochrome c oxidase subunit 7a1 (*Cox7a1*), another cold-inducible brown adipocyte marker [46,47] and part of complex IV of the mitochondrial respiratory chain, was also reduced by RetSat depletion in mature brown adipocytes (Figure 3B). Notably, retroviral overexpression of RetSat in undifferentiated iBACs, which does not affect pioglitazone-driven differentiation (Figure 2H, right panel), also did not affect thermogenic gene expression (Supplemental Fig. S4A) or UCP1 protein induction by isoproterenol in mature brown adipocytes (Supplemental Fig. S4B). This was despite validating RetSat overexpression in brown adipocytes as being functional, evidenced by the known increase in ROS production upon peroxide exposure [12] (Supplemental Fig. S4C and its densitometric analysis in S3D), suggesting that RetSat is required but not sufficient to drive adequate thermogenic gene and protein expression in these cells.

We next analyzed RetSat's function in *in vitro*-differentiated primary brown adipocytes derived from mice with floxed *RetSat* alleles, where exons 2 and 3, encoding the dinucleotide-binding motif essential for its enzymatic activity [2], are flanked by loxP sites [36]. Adenoviral expression of Cre recombinase in differentiated brown adipocytes efficiently deleted RETSAT protein expression (Figure 3C). Similar to our observations in differentiatied iBACs, RetSat deletion reduced expression and isoproterenol-inducibility of *Ucp1* and *Elovl3* mRNA in differentiated primary brown adipocytes (Figure 3D). Moreover, RetSat deletion reduced oxygen consumption rate (OCR) of primary brown adipocytes and providing evidence for impaired mitochondrial function (Figure 3E, left and right panel).

### 3.4 RetSat deletion in BAT impairs acute cold tolerance of mice

**Figure 4:**
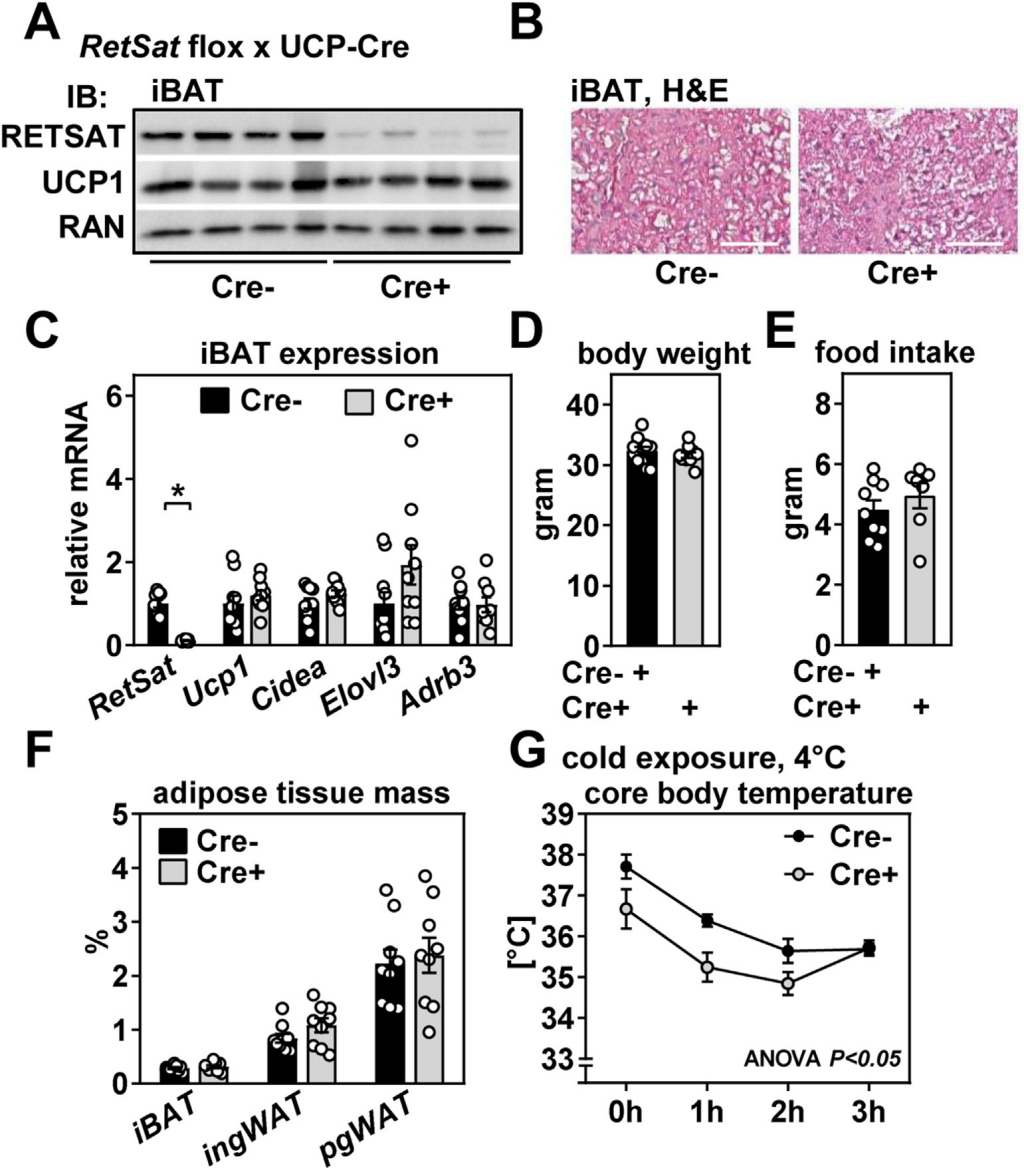
**RetSat deletion in brown adipose tissue of mice impairs acute cold tolerance.** A) Protein expression of RETSAT and UCP1 in interscapular brown adipose tissue (iBAT) of 3 months old, male mice of the indicated genotypes was determined by immunoblotting, RAN served as loading control. B) iBAT morphology of Cre- and Cre+ mice was analyzed by hematoxylin & eosin staining (H&E, scale bar = 50 μm). C) mRNA expression of indicated genes in mice described in A) was determined by qPCR. D) Body weights and E) 24 h food intake of mice described in A). F) Relative adipose tissue mass of iBAT and inguinal/perigonadal white adipose tissue (ing/pgWAT) in Cre- and Cre+ mice. G) Cre- and Cre+ mice were exposed to 4 °C and core body temperature determined by a rectal probe at indicated time points (n = 9,7). In C) (n = 10,10), D) (n = 11,10), E) (n = 9,7), and F) (n = 9,9), data are presented as individual data points and mean ± sem, ∗*P* < 0.05.

To dissect RetSat's function in BAT *in vivo*, we crossed mice with floxed RetSat alleles [36] with mice that express Cre under the control of the *Ucp1* promoter [37]. Cre-driven recombination efficiently deleted RETSAT protein in iBAT (Figure 4A), but not in ing/pgWAT or liver (Supplemental Figs. S5A–C). Residual RETSAT expression in iBAT upon recombination likely reflects its expression in *Ucp1*-negative cells present in this tissue. Mice were born at the expected Mendelian ratio and appeared indistinguishable from their control littermates (data not shown). Surprisingly, and in contrast to RetSat depletion in brown adipocytes, there was no discernible difference in UCP1 protein abundance in iBAT between Cre negative (Cre-) and Cre positive (Cre+) mice (Figure 4A) kept at 21 °C. iBAT morphology, both basally or upon activation by repeated β3A injections, also appeared comparable (Figure 4B and Supplemental Fig. S6). Thermogenic gene expression in iBAT, body weights, food intake, and relative fat depot mass did not differ between Cre- and Cre+ mice (Figure 4C–F). However, exposing single-housed mice to 4 °C showed a significant, but transient cold intolerance of Cre+ mice, presenting itself with lower core body temperatures before and during early time points that disappeared after 3 h (Figure 4G). During longer exposure to 4 °C for up to 14 days, Cre+ mice maintained their body temperature similarly well as Cre-mice (Supplemental Fig. S7). We conclude that *Ucp1*-Cre driven deletion of RetSat does not result in major differences in morphology or thermogenic gene expression of iBAT, but leads to a transient intolerance during an acute cold challenge that appears to be compensated at later time points. Thus, RetSat is involved in the immediate cold response of BAT but less relevant for the long-term adaptations to cold. Regarding glucose metabolism, *Ucp1*-Cre driven deletion of RetSat had no effect on blood glucose concentrations in *ad libitum*-fed mice but increased blood glucose levels in mice fasted for 16 h (Supplemental Fig. S8).

Challenging male mice with HFD for 12 weeks did not induce differences in body weight, weight gain, body composition, or relative fat depot mass between Cre- and Cre+ mice (Supplemental Figs. S9A–C). In accordance, glucose tolerance was slightly impaired in Cre+ mice (Supplemental Fig. S9D, left and right panel) whereas an insulin injection lowered blood glucose concentrations to a similar extent in both genotypes (Supplemental Fig. S9E, left and right panel). However, after a 16-hour fast, Cre+ mice showed elevated blood glucose levels (Supplemental Fig. S9F). iBAT ROS levels, analyzed by determining tissue malondialdehyde (MDA) content, were not affected by RetSat deletion in both NC-and HFD-fed mice (Supplemental Figs. S10A and S10B). Taken together, BAT-specific deletion of RetSat in mice does not elicit a major metabolic phenotype, even when fed HFD, except a mild impairment of glucose homeostasis with increased blood glucose upon fasting.

### 3.5 RetSat deletion in BAT downregulates expression of mitochondrially encoded genes

**Figure 5:**
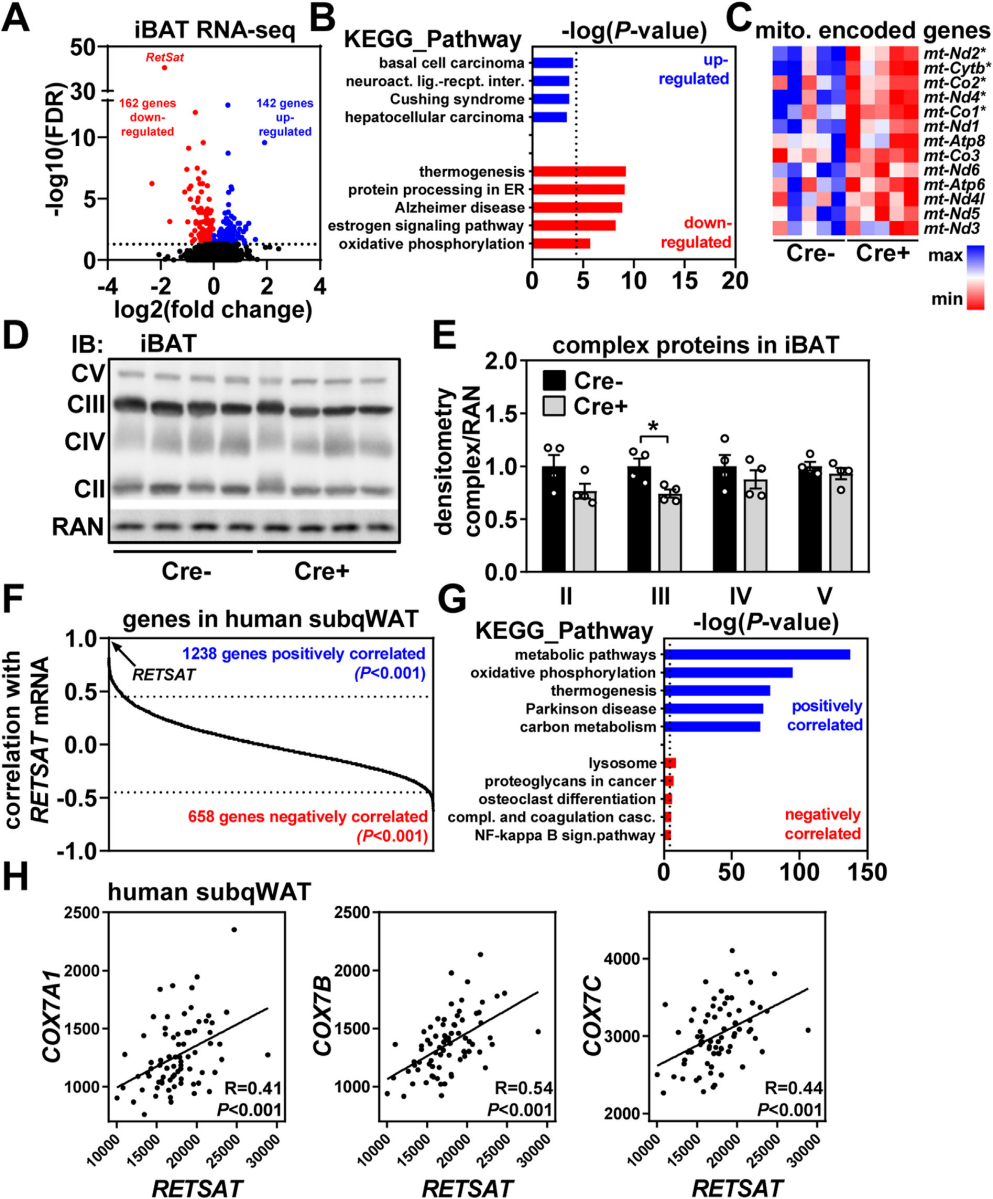
**RetSat regulates mitochondrially encoded genes in murine brown adipose tissue and is correlated with genes related to oxidative phosphorylation and thermogenesis in white adipose tissue of human subjects.** A) Significantly (FDR<5%) upregulated (blute dots) and downregulated (red dots) genes in interscapular brown adipose tissue (iBAT) of Cre+ mice (n = 5,5). B) KEGG pathway enrichment in up- and downregulated genes in iBAT of Cre+ mice. C) Heatmap of all detected mitochondrially encoded genes in iBAT of Cre- and Cre+ mice. D) iBAT protein expression of electron transport chain complexes II-V in Cre- and Cre+ mice were determined by immunoblotting. RAN served as loading control. E) Densitometric analysis of immunoblots shown in D). F) Positively and negatively correlated genes with *RetSat* mRNA expression in subcutaneous white adipose tissue (subqWAT) of human subjects. G) KEGG pathway enrichment in genes positively or negatively-correlated with *RetSat* mRNA in human subqWAT. H) Correlation of *RetSat* mRNA with the expression of COX subunits in human subqWAT, n = 75. In C), ∗FDR<5%, in E) (n = 4,4), data are presented as individual data points and mean ± sem, ∗*P* < 0.05.

Transcriptional consequences of RetSat deletion in iBAT were analyzed by RNA sequencing (RNAseq) and yielded 304 differentially expressed genes (FDR<5%) (Figure 5A). Enriched KEGG pathways among upregulated genes did not reach statistical significance, downregulated genes clustered, among others, to *thermogenesis* and *oxidative phosphorylation* (Figure 5B). The largest contributors to both pathways were mitochondrial DNA-encoded genes such as *mt-Nd2*, *mt-Nd4*, *mt-Cytb*, *mt-Co1*, and *mt-Co2*. In fact, visualizing all protein-encoding genes of mitochondrial DNA using a heatmap showed a rather consistent pattern of downregulation (Figure 5C). We therefore probed iBAT protein for electron transport chain complexes and found overall reduced abundance in Cre+ mice (Figure 5D). Thus, RetSat deletion in BAT reduces expression of mitochondrial DNA-encoded genes and the abundance of electron transport chain complex proteins.

### 3.6 *RETSAT* mRNA expression in human subqWAT correlates with genes related to mitochondrial function

Given RetSat's impact on mitochondrial gene expression, we analyzed previously reported RNAseq expression profiles of subqWAT biopsies from 75 overweight and obese subjects [41]. With a cut-off of *P* < 0.001, 1238 genes were positively, and 658 genes negatively correlated with *RETSAT* mRNA expression, respectively (Figure 5F). Very little pathway enrichment was detected in negatively correlating genes, contrasted by highly significant clustering of positively correlating genes to KEGG pathways like *oxidative phosphorylation* and *thermogenesis* (Figure 5G). Among these were several COX subunits including *COX7A1* (Figure 5H). We conclude that in human subqWAT, *RETSAT* mRNA expression correlates with gene expression signatures related to mitochondrial function.

### 3.7 RetSat deletion in BAT/depletion in adipocytes downregulates genes involved in protein folding

**Figure 6:**
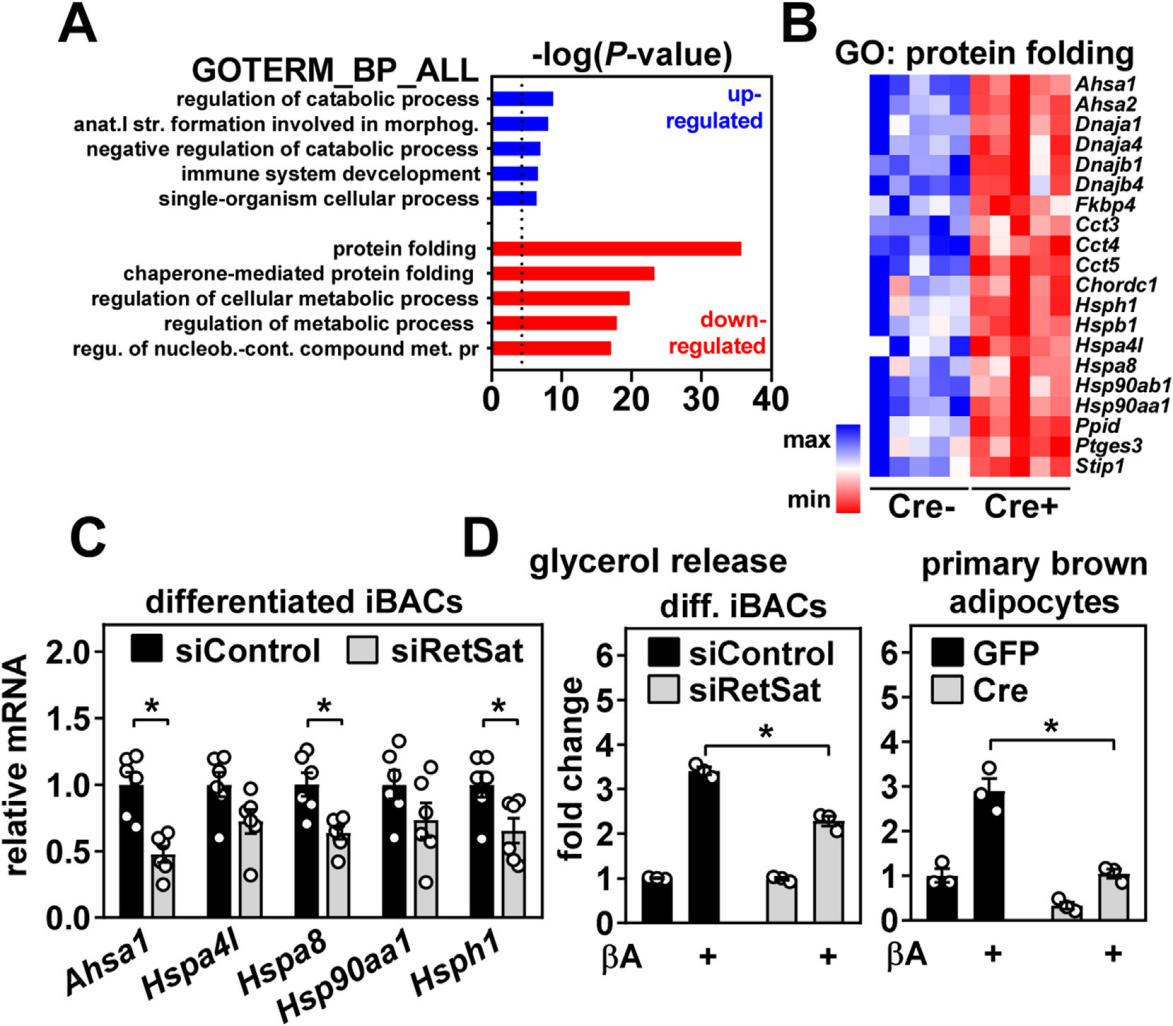
**RetSat deletion in brown adipose tissue downregulates genes involved in protein folding and its depletion in brown adipocytes impairs lipolysis.** A) Enriched gene ontology (GO) terms of biological process (BP) in up- and downregulated genes (FDR<5%) in interscapular brown adipose tissue (iBAT) of Cre+ mice. B) Downregulated genes (FDR<5%) in iBAT of Cre+ mice associates with the GO term protein folding, n = 5,5. C) Expression of indicated genes in differentiated iBACs in the presence of 10 μM of the pan-β-adrenergic receptor agonist isoproterenol for 4 h was determined by qPCR. D) Differentiated iBACs (left panel) and differentiated primary brown adipocytes (right panel) were depleted/deleted of *RetSat*, stimulated with 10 μM of the pan-β-adrenergic receptor agonist (βA) isoproterenol for 4 h, and glycerol release determined. In C) (n = 6,6) and D) (n = 3,3), data are presented as individual data points and mean ± sem, ∗*P* < 0.05.

We found that known RA-responsive genes [[48], [49], [50]] or transcriptional targets of ChREBP [51] were not regulated by RetSat deletion in BAT (Supplemental Figs. S11A and S11B), suggesting that these pathways, although linked previously to RetSat [6,9], were unlikely to contribute to the observed effects. However, besides *thermogenesis* and *oxidative phosphorylation*, downregulated genes in iBAT of Cre+ mice also clustered to the KEGG pathway *protein processing in ER* (Figure 5B), comprising a variety of heat shock protein (HSP) members of the Hsp40, -70, −90, and −110 families. Accordingly, there was a striking enrichment of the GO terms *protein folding* and *chaperone mediated protein folding* in genes that were significantly downregulated by RetSat deletion (Figure 6A,B). Reduced HSP and co-chaperone expression was validated by qPCR (Supplemental Fig. S12A). Of known transcription factors that control expression of HSP and that of other proteins related to protein folding and the unfolded protein response (UPR) [21,52], only CCAAT-enhancer-binding protein β (*Cebpb*) was regulated (FDR<5%) and expressed at lower levels in iBAT of Cre+ mice (Supplemental Fig. S12B), suggesting an involvement of Cebpb in these regulations. Interestingly, pathways related to the unfolded protein response and ER-related protein folding are known to be induced in BAT upon cold exposure and β-adrenergic stimulation [[53], [54], [55]]. Reduced HSP and co-chaperone expression was also observed in brown adipocytes depleted of RetSat (Figure 6C), implying an adipocyte-autonomous regulation.

### 3.8 RetSat depletion in adipocytes impairs lipolysis

RetSat depletion in brown and beige adipocytes led to a concerted reduction of thermogenic gene expression and its deletion in BAT of mice resulted in >300 significantly regulated genes, clustering to pathways such as to *thermogenesis*, *oxidative phosphorylation* and *protein folding*. To explain these robust transcriptional effects, we hypothesized that loss of RetSat may impair lipolysis, a major regulator of gene transcription related to above pathways in adipocytes [21,23]. Indeed, glycerol release of βA-stimulated iBACs and primary brown adipocytes was reduced upon depletion or deletion of RetSat (Figure 6D). A similar reduction of glycerol release was observed in stimulated 3T3-L1 adipocytes treated with RetSat-targeting siRNA and accompanied by lower non-esterified fatty acid (NEFA) concentrations in the culture media (Supplemental Fig. S13A). Furthermore, RetSat depletion in 3T3-L1 adipocytes also reduced glycerol and NEFA release induced by the cell-permeable cAMP analogue dibutyryl-cAMP (Supplemental Fig. S13B), showing that the lipolytic defect is downstream of β-adrenergic receptor activation and cAMP generation. Retroviral overexpression of RetSat in iBACs had no effect on glycerol release (Supplemental Fig. S14), which is consistent with lacking effects on thermogenic gene expression in these cells (Supplemental Fig. S4A) and suggesting that RetSat is required but not sufficient for the maximal stimulation of lipolysis by β-adrenergic stimulation. In mice, deletion of RetSat in BAT had no effects on serum concentrations of glycerol and NEFA in *ad libitum*-fed mice on NC (Supplemental Fig. S15A) or HFD (Supplemental Fig. S15B), suggesting that a potential defect in BAT lipolysis by deletion of RetSat is not affecting steady state levels of these metabolites in the circulation.

## 4 Discussion

Previous studies implicated RetSat in the differentiation of white adipocytes [2]. Here we report a similar, but more subtle induction of brown adipocyte differentiation by RetSat overexpression in precursor cells *in vitro*. Besides this finding, we discovered that RetSat is required for adequate expression of thermogenic genes in brown adipocytes, in particular upon β-adrenergic stimulation. In fact, RetSat expression itself is regulated by β-adrenergic signaling; it is induced in WAT and BAT of mice as well as in white and brown adipocytes after repeated/prolonged βA exposure, but downregulated in adipocytes by an acute βA exposure of 4 h. Thus, adipocyte RetSat expression is under β-adrenergic control, which likely procures the increase in RetSat expression in BAT and WAT of mice exposed to cold and in BAT of obese, HFD-fed mice. Whether this regulation is due to cyclic nucleotide-activated transcription factors like ‘cAMP response element-binding protein’, or indirectly by involving lipolysis-derived fatty acids that can active certain PPARs [25], or both, is currently unknown and will require further scrutiny.

Besides thermogenic genes like *Ucp1*, *Elovl3*, and *Cidea*, RetSat depletion also reduced expression of *Cox7a1* that encodes a complex IV subunit of the respiratory chain in mitochondria. Accordingly, RetSat depletion lowered oxygen consumption of primary brown adipocytes. Regulation of other yet unidentified genes and metabolites by RetSat depletion is likely to contribute to this phenotype.

Considering these major transcriptional responses in brown adipocytes, we were surprised that BAT-specific deletion of RetSat in mice had no discernible effect on the expression of these thermogenic genes *in vivo*. The reasons for this discrepancy are uncertain but may include a compensational activation of other drivers of thermogenic gene expression, such as certain transcription factors or sympathetic signals, to warrant BAT homeostasis. Moreover, since Cre expression driven by the *Ucp1* promoter in brown adipocytes was constitutive, effects of an acute RetSat deletion in BAT may differ. However, lower UCP1 protein expression in brown adipocytes upon RetSat depletion was detectable for at least 7 days, arguing against a transient effect and rapid counter-regulation. Despite lacking effects on genes like *Ucp1*, *Cidea*, *Elovl3*, and *Adrb3*, overall expression of mitochondrially-encoded genes in iBAT was decreased, as were genes related to the GO terms ‘protein processing in the ER’ and ‘protein folding’, including many HSP members. These changes may indeed point towards impaired BAT plasticity since the majority of the latter are induced by exposing mice to cold, including *Ahsa1, Dnaja1, Dnajb1, Hsp90aa1, Hsp90ab1, Hspa8, Hspa4l, Hsph1,* and *Cct3* [53]*.* Phenotypically, mice with BAT-specific RetSat deletion showed a transient intolerance to an acute cold challenge but appeared otherwise metabolically unremarkable, except increased fasting blood glucose levels. Whether deleting RetSat in all fat depots instead of BAT alone induces a more severe metabolic phenotype would be worth addressing in future experiments.

Although RetSat depletion in cells reduced peroxide-induced ROS production [10,12], there was no reduction in ROS production in iBAT that lacks RetSat. Interestingly, global deletion of RetSat decreased ROS production in liver, but not WAT [12], suggesting that compensational effects may depend on the specific tissue.

At this point, we have no indication that RetSat-dependent alterations in RA signaling [9] or ChREBP activation [6] contribute to the observed transcriptional effects in BAT. Instead, we focused on lipolysis, since it is known to mediate some of the transcriptional consequences of β-adrenergic stimulation in adipocytes [21,25,26]. Accordingly, it was shown that genetic loss-of-function of adipose triglyceride lipase (Atgl) or of its coactivator α/β hydrolase domain containing 5 (Abhd5) regulates gene expression in brown adipose tissue [[56], [57], [58], [59]]. RetSat depletion in brown adipocytes reduced glycerol release, indicating that RetSat expression is indeed required for full lipolytic activity upon βA exposure. Thus, impaired lipolysis due to RetSat depletion could explain reduced thermogenic gene expression and mitochondrial respiration in brown adipocytes. Although we cannot exclude that RetSat depletion impairs β-adrenergic signaling, reduced expression of thermogenic genes in the absence of pharmacological βA and lower cAMP analogue-induced lipolysis render this possibility less likely. Furthermore, the robust expression of RetSat in lipolytic tissues [1,2,6], its β-adrenergic regulation, fasting-inducibility in many tissues [5], and increased adiposity [15] and hepatic triglycerides in mice with global RetSat deletion [12] are all compatible with RetSat modulating lipolysis.

Regarding thermogenic gene expression in BAT, one could hypothesize that expression of mitochondrially-encoded genes and genes related to protein folding may respond to impaired lipolysis more sensitively than *Ucp1*, *Cidea*, *Elovl3*, and *Adrb3*, explaining why the latter genes were not affected by loss of RetSat *in vivo*. Also circulating NEFA levels were unchanged by RetSat deletion in BAT, consistent with the notion that lipolysis in WAT rather than BAT contributes to serum NEFA. Interestingly, circulating NEFA were reduced by an acute RetSat depletion in liver of HFD-fed mice [6]. Thus, RetSat may affect lipolysis in a context-dependent manner and, depending on the organ targeted for deletion, not necessarily affect circulating NEFA levels. RetSat deletion in mice would also be expected to result in less pronounced phenotypes than those upon the deletion of lipases or coregulators (e.g. Abhd5 or G0/G1 switch gene 2 [60]), since its link to lipolysis is most likely indirect. Nevertheless, other mechanisms than reduced lipolysis may contribute to the effects of RetSat loss-of-function. How exactly the depletion of ER-localized RetSat in adipocytes interferes with the lipolytic pathway is still unclear, which is a limitation of our study.

We would speculate that RetSat's function in brown adipocytes and mouse BAT depends on its oxidoreductase activity. In white adipocyte precursor cells, RetSat overexpression enhanced differentiation only when enzymatically active [2]. In respect to the enzymatic reaction catalyzed by RetSat in brown adipocytes and BAT, we have no indication that RetSat generates 13,14-dihydroretinol while depleting retinol. This is due to our observation that expression of RA-responsive genes in iBAT, which might be induced if the ratio of retinoids (potent RAR activators) to dihydroretinoids (less potent RAR activators [8]) upon the loss of RetSat increases, was not affected by RetSat deletion. Moreover, in both white adipocyte differentiation [2] and glucose-induced gene expression in hepatocytes [6], providing 13,14-dihydroretinol to RetSat-depleted cells could not rescue the observed defects. Finally, 13,14-dihydroretinol could not be detected in RetSat-overexpressing adipocytes [2]. Therefore, the most likely scenario is that RetSat catalyzes a yet unknown reaction in BAT that underlies the observed effects upon RetSat deletion.

## 5 Conclusions

Our findings vastly expand the understanding of RetSat's regulation and function in brown adipocytes and its relevance for non-shivering thermogenesis and BAT physiology in mice. Although insights into alternative enzymatic reactions are still lacking, implicating RetSat in the regulation of lipolysis may allow for more specific approaches to unravel its enzymatic activities and warranting further research.

## Author's contributions

C.L., M.F.K., S.D., R.E.F., N.Y., Y.M., S.W., S.G., M.So., S.J.W., K.M.P., and D.D., performed experiments. C.L., L.S., K.M., H.S, T.J.S. and M.S. handled, evaluated, or discussed data and edited the manuscript. C.L. and M.S. wrote the original manuscript and are the guarantors of this work. All authors reviewed and commented on the article.

## CRediT authorship contribution statement

**Chen Li:** Data curation, Methodology, Writing – review & editing. **Marie F. Kiefer:** Data curation. **Sarah Dittrich:** Data curation. **Roberto E. Flores:** Data curation, Methodology. **Yueming Meng:** Data curation. **Na Yang:** Data curation. **Sascha Wulff:** Data curation. **Sabrina Gohlke:** Data curation. **Manuela Sommerfeld:** Data curation. **Sylvia J. Wowro:** Data curation, Funding acquisition. **Konstantin M. Petricek:** Data curation. **Dominic Dürbeck:** Data curation. **Leonard Spranger:** Data curation. **Knut Mai:** Data curation, Supervision. **Holger Scholz:** Conceptualization, Supervision, Writing – review & editing. **Tim J. Schulz:** Conceptualization, Data curation, Supervision, Writing – review & editing. **Michael Schupp:** Conceptualization, Funding acquisition, Methodology, Supervision, Writing – original draft, Writing – review & editing.

## Declaration of competing interest

The authors declare that they have no known competing financial interests or personal relationships that could have appeared to influence the work reported in this paper.

## Data Availability

Data will be made available on request.
