# Supplementary material for: Adipose retinol saturase is regulated by β-adrenergic signaling and its deletion impairs lipolysis in adipocytes and acute cold tolerance in mice
Source: Mol Metab. 2023 Dec 19;79:101855. doi: 10.1016/j.molmet.2023.101855 (PMC10784691; doi:10.1016/j.molmet.2023.101855)
Supplement: Multimedia component 1 [file mmc1.pdf]

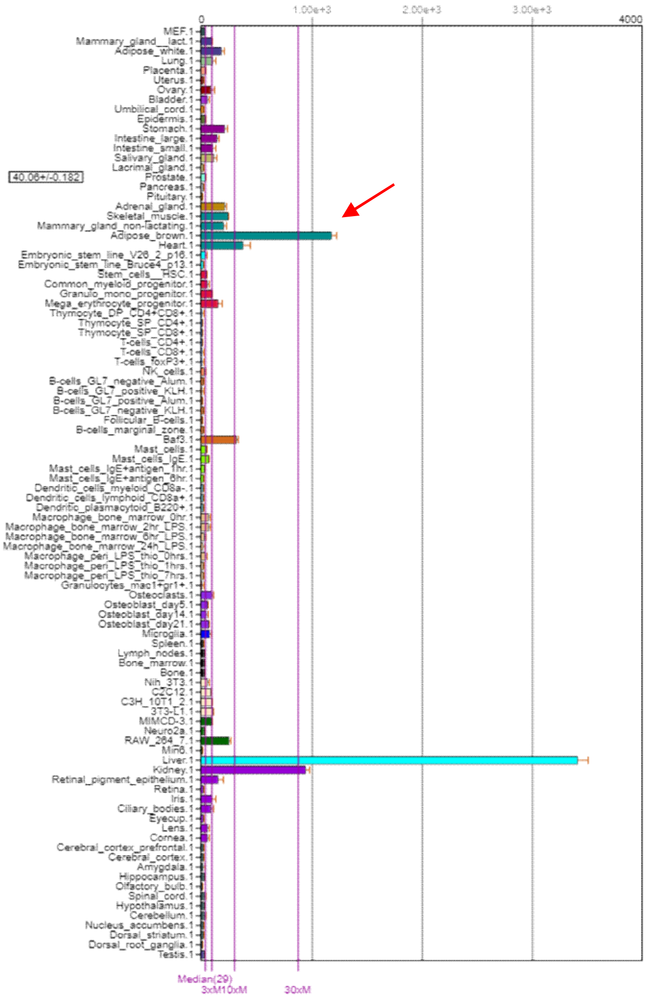

Supplemental Figure S1. Tissue distribution of *RetSat* expression in mice. Gene expression profile of murine *RetSat* from BioGPS.org (probeset 1424716_at).

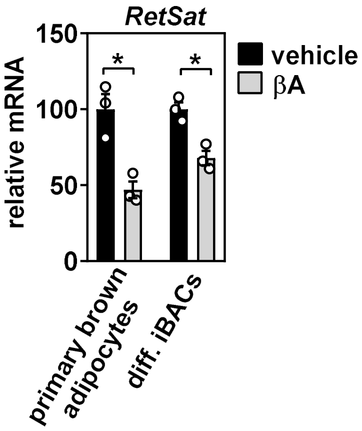

Supplemental Figure S2. Short-time exposure to β-agonist decreases *RetSat* expression in brown adipocytes. Differentiated primary brown adipocytes or differentiated immortalized brown adipogenic cells (iBACs) were incubated the pan-β-adrenergic receptor agonist (βA) isoproterenol for 4 hours and mRNA expression of *RetSat* determined by qPCR, n=3,3. Data are presented as individual data points and mean±sem, *P<0.05.

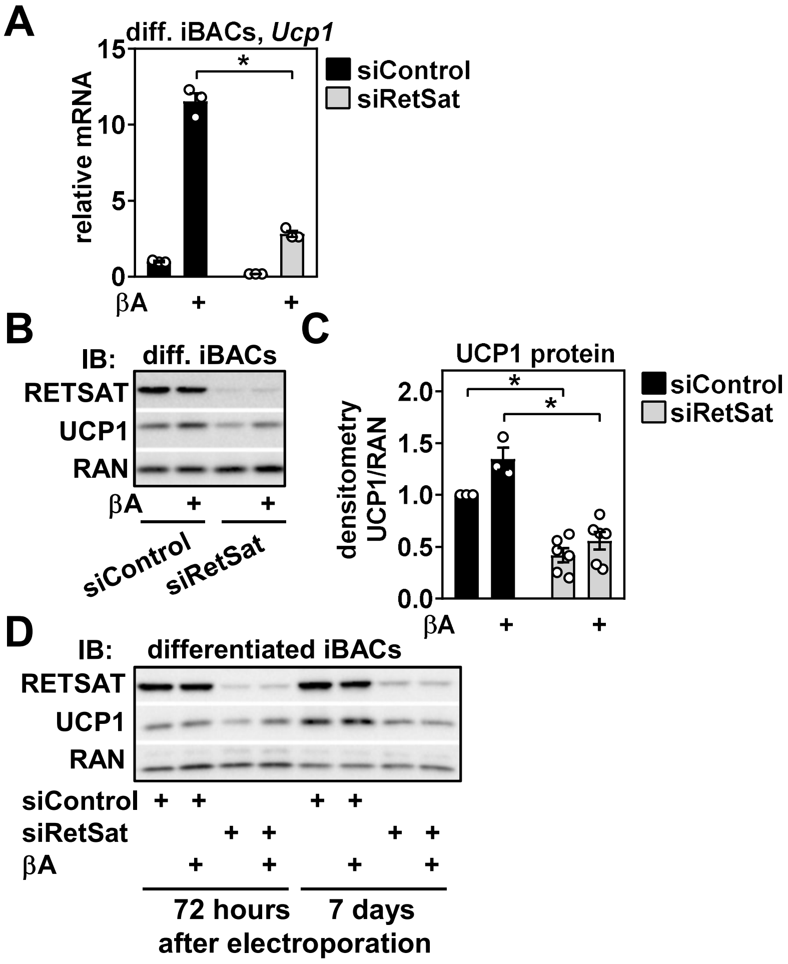

Supplemental Figure S3. RetSat depletion in brown adipocytes reduced maximal induction of Ucp1 expression upon β-adrenergic stimulation. Differentiated immortalized brown adipogenic cells (iBACs) were electroporated with RetSat siRNA. After 72 hours, cells were exposed to the pan-β-adrenergic receptor agonist isoproterenol for 4 hours and A) *Ucp1* mRNA and B) UCP1 protein expression determined. In B), RAN protein served as loading control. C)) Densitometric analysis of UCP1 protein shown in B). D) Cells were electroporated as described in A) and after 72 hours or 7 days treated as indicated and UCP1 protein expression analyzed by immunoblotting. RAN served as loading control. In A (n=3,3) and C (n=3,3,6,6), data are presented as individual data points and mean±sem, *P<0.05.

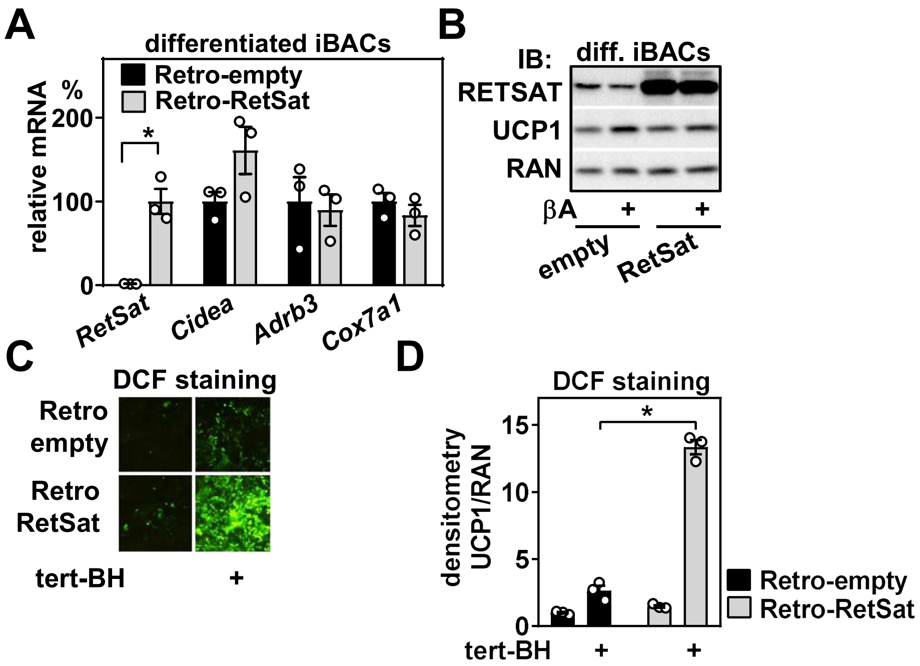

Supplemental Figure S4. RetSat gain-of-function does not increase the expression of thermogenic genes and UCP1 protein in brown adipocytes. Immortalized brown adipogenic cells (iBACs) were infected with empty- or RetSat-encoding retroviruses and differentiated in the presence of pioglitazone. After differentiation, A) mRNA expression of indicated genes was determined by qPCR, and B) RETSAT and UCP1 protein analyzed by immunoblotting. RAN served as loading control. C) Functionality of ectopic RETSAT expression in differentiated iBACs was validated by elevated reactive oxygen species, detected by dichlorodihydrofluorescein-diacetate (DCF) staining, after stimulation with *tert*-butyl hydroperoxide (tert-BH) as peroxide stressor. D) Densitometric analysis of DCF staining shown in C). In A) (n=3,3) and D) (n=3,3), data are presented as individual data points and mean±sem, *P<0.05

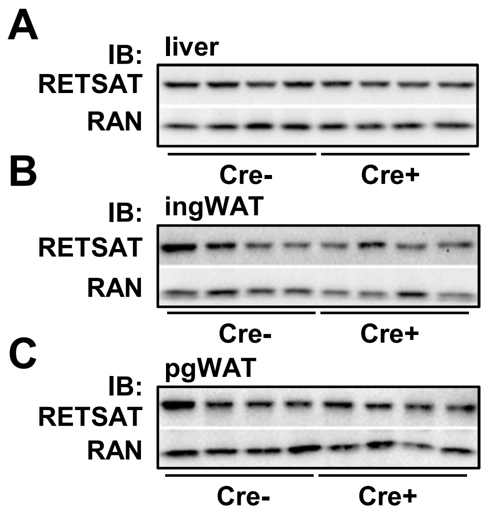

Supplemental Figure S5. UCP1-Cre mediated RetSat ko is brown adipose tissue specific. Protein expression of RETSAT in Cre- and Cre+ mice in A) liver, B) inguinal-, and C) perigonadal white adipose tissue (ing/pgWAT) was determined by immunoblotting. RAN served as loading control.

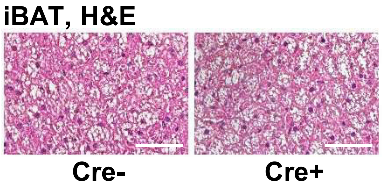

Supplemental Figure S6. RetSat deletion in BAT has no effect on tissue morphology after repeated β3-agonist injections. Brown adipose tissue (BAT) morphology of Cre- and Cre+ mice that were intraperitoneally injected for 10 days with the β3-agonist CL316,243 was analyzed by hematoxylin & eosin staining (H&E, scale bar=50 µm).

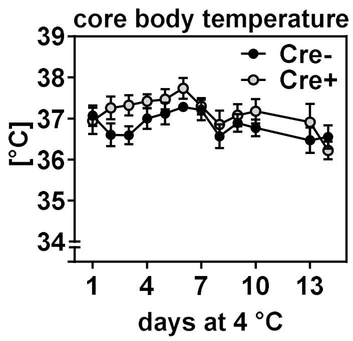

Supplemental Figure S7. RetSat deletion in brown adipose tissue has no effect on long-term cold tolerance. Cre- and Cre+ mice were exposed to 4 °C and core body temperature determined by a rectal probe at indicated times points (n=10,9).

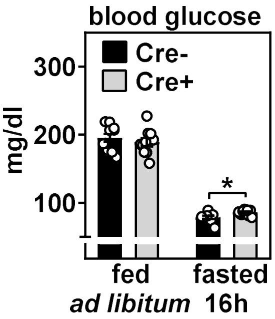

Supplemental Figure S8. RetSat deletion in BAT increases fasted blood glucose conentrations. Blood glucose concentration in *ad libitum*-fed or 16 hours fasted mice of indicated genotypes on normal chow was determined (n=10,10). Data are presented as individual data points and mean±sem, **P*<0.05.

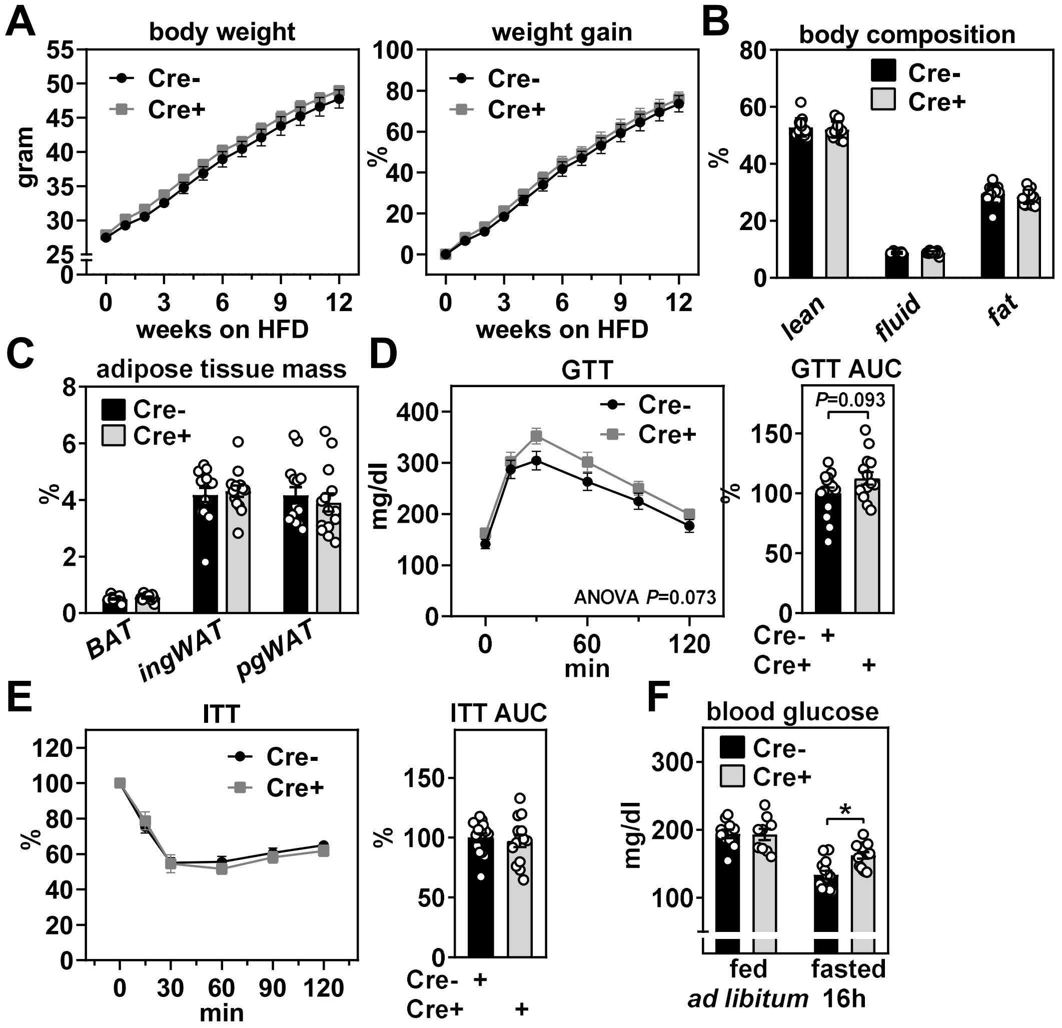

Supplemental Figure S9. RetSat deletion in brown adipose tissue of high fat-diet fed mice. Male mice of the indicated genotypes were fed high fat-diet (HFD, 60% kcal/fat) for 12 weeks, starting at an age of 10 weeks, and A) body weights and weight gain determined (n=14,14). B) Body composition of Cre- and Cre+ mice after HFD-feeding was determined by NMR. C) Adipose tissue mass of indicated depots was weighed in Cre- and Cre+ mice fed HFD. D) Glucose and E) insulin tolerance of HFD-fed mice with indicated genotype. F) Blood glucose concentration in *ad libitum*-fed or 16 hours fasted mice on HFD was determined. In B), C), D), E) (all n=14,14), and F) (n=10,9,14,14), data are presented as individual data points and mean±sem, **P*<0.05 vs. Cre- mice.

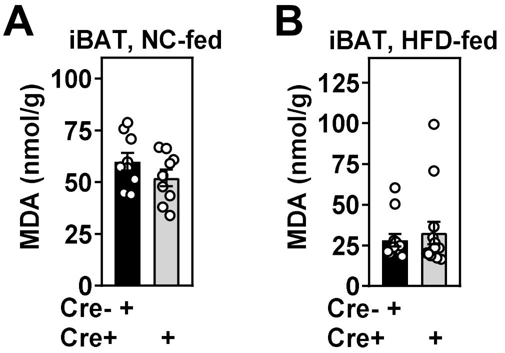

Supplemental Figure S10. RetSat deletion in brown adipose tissue (BAT) does not alter tissue abundance of malondialdehyde (MDA). MDA abundance in interscapular BAT (iBAT) of male mice fed normal chow (NC) or high-fat diet (HFD). In A) (n=9,9) and B) (n=12,13), data are presented as individual data points and mean±sem.

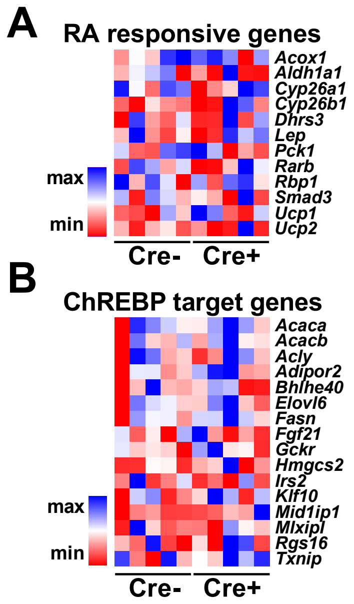

Supplemental Figure S11. RetSat deletion in brown adipose tissue does not affect retinoic acid-responsive or ChREBP target gene expression. Expression of A) retinoic acid (RA) responsive genes and B) ChREBP target genes in interscapular brown adipose tissue of male Cre- and Cre+ mice.

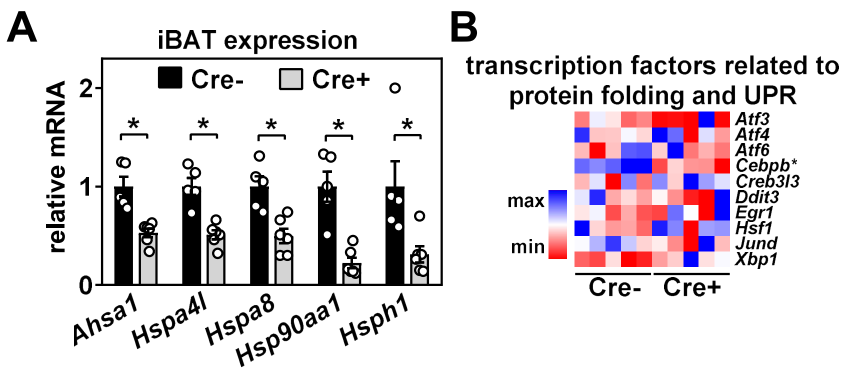

Supplemental Figure S12. RetSat deletion in brown adipose tissue and reduces expression of genes involved in protein folding. A) Expression of indicated genes in interscapular brown adipose tissue (iBAT) of Cre- and Cre+ mice. B) Expression of known transcriptional regulators of genes involved in protein folding and the unfolded protein response in iBAT of male Cre- and Cre+ mice. In A) (n=5,6), data are presented as individual data points and mean±sem, *P<0.05.

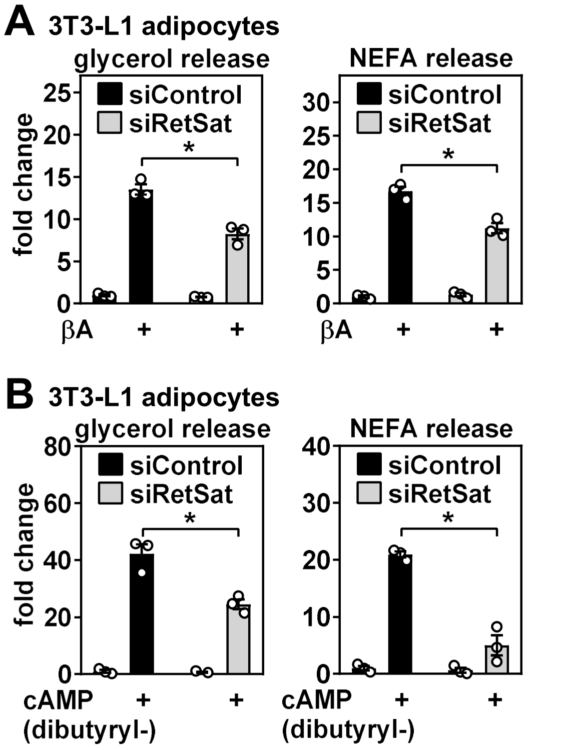

Supplemental Figure S13. RetSat depletion in 3T3-L1 adipocytes impairs lipolysis. Differentiated 3T3-L1 adipocytes were depleted of *RetSat*, stimulated with A) 10 μM of the pan-β-adrenergic receptor agonist (βA) isoproterenol or B) 1 mM of dibutyryl-cAMP for 4 hours, and the release of glycerol (left panel) and non-esterified fatty acids (NEFA)(right panel) determined. In A) (n=3,3) and B) (n=3,3), data are presented as individual data points and mean±sem, **P*<0.05.

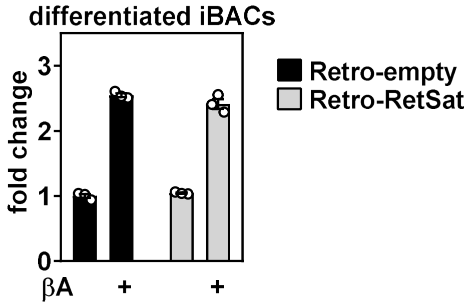

Supplemental Figure S14. RetSat gain-of-function does not affect glycerol release of brown adipocytes. Immortalized brown adipogenic cells (iBACs) were infected with empty- or RetSat-encoding retroviruses and differentiated in the presence of pioglitazone. After differentiation, adipocytes were stimulated with 10 µM of the pan-β-adrenergic receptor agonist (βA) isoproterenol for 4 hours, and glycerol release determined (n=3,3). Data are presented as individual data points and mean±sem

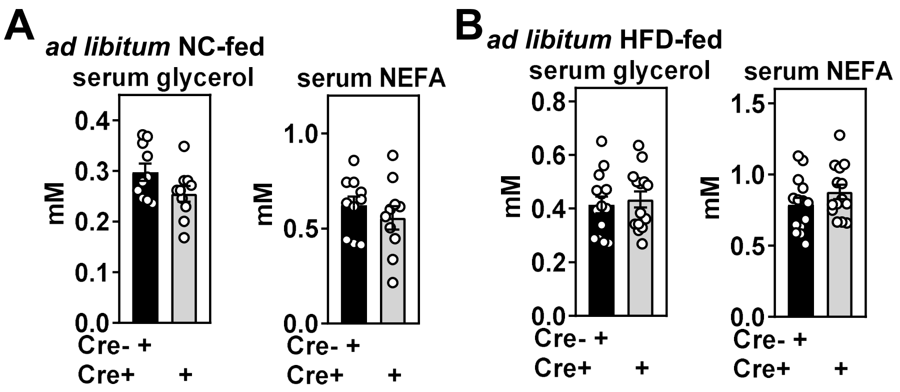

Supplemental Figure S15. RetSat deletion in brown adipose tissue of mice does not alter circulating glycerol and NEFA concentrations. Serum glycerol and NEFA concentrations in *ad libitum*-fed mice on A) normal chow (NC) or B) high fat-diet (HFD). In A) (n=10,10) and B) (n=14,14), data are presented as individual data points and mean±sem.

Supplemental Tables

Table S1. siRNA oligonucleotides sequences (5'-3')

target	sense	antisense
siControl (non-targeting)	UAGCGACUAAACACAUCAAUU	UUGAUGUGUUUAGUCGCUAUU
siRetSat_1	UCAGCCGAGUACCAGAGAAUU	UUCUCUGGUACUCGGCUGAUU
siRetSat_2	GCUCAAAGGUCAAGGCACAUU	UGUGCCUUGACCUUUGAGCUU

Table S2. (q)PCR oligonucleotide sequences (5'-3')

gene/region	usage	forward	reverse
RetSat flox	gen.typ.	CTCCTTTTCTGAGGCTGGTG	AAGGCAGACCTTTCTTTTAAGG
Ucp1-Cre	gen.typ.	CGATGCAACGAGTGATGAGGTTC	GCACGTTCACCGGCATCAAC
m18s	qPCR	AAACGGCTACCACATCCAAG	GGCCTCGAAAGAGTCCTGTA
mAhsa1	qPCR	GCTGGTAGCGGTGCGATAAG	ATCTCTCTCTGTCCAGTGCCA
mAdrb3	qPCR	GGCCCTCTCTAGTTCCCAG	TAGCCATCAAACCTGTTGAGC
mCidea	qPCR	AGGGACACCACGCATTTCAT	CCGATTTCTTTGGTTGCTTG
mCox7a1	qPCR	AAAACCGTGTGGCAGAGAAG	CAGCGTCATGGTCAGTCTGT
Cre	qPCR	GCTTGCATGATCTCCGGTAT	ATACCTGGCCTGGTCTGGA
mElovl3	qPCR	GTGGTCGTCTATCTGTTGCTCA	GCCAGGAAGAAGGACCAGAG
mFabp4	qPCR	TGGAAGACAGCTCCTCCTCG	AATCCCCATTTACGCTGATGATC
mHsp90aa1	qPCR	CAATTCATCGGACGCTCTGG	TCAGGGTTCGGTCCTGTTTG
mHspa4l	qPCR	TGCAAACGGAGAGGATCAGG	CCAGGTACCGCACCTTAACA
mHspa8	qPCR	CCTGGTGTACTCATTCAGGTGT	TGCCTGTGAGCTCGAACTTT
mHsph1	qPCR	ATGACATGACGGCTGTTGCT	CCCATGTCAACAAACACCACC
mPparg2	qPCR	TGGGTGAAACTCTGGGAGATTC	GAGAGGTCCACAGAGCTGATTCC
mPrdm16	qPCR	TCTGCCACAAGTCCTACACG	GAACATCTGCCCACAGTCCT
mRetSat	qPCR	GCGGCTGTTGTCATACCTTT	CCAAGATAAAACGGCCAATG
mRplp0	qPCR	TCATCCAGCAGGTGTTTGACA	GGCACCGAGGCAACAGTT
mUcp1	qPCR	GGGCCCTTGTAAACAACAAA	ACTGGAGAGGCCAGGAGTGT

Table S3. Antibodies

antibody	product	application
PPARγ	Santa Cruz, sc-7273 (E8)	1:200 in 2.5% skim milk
OXPHOS (total)	Abcam, ab110413	1:5000 in 4% skim milk
RAN	BD Biosciences, #610341	1:5000 in 4% skim milk
RETSAT	Sigma HPA046513	1:1000 in 4% skim milk
TUBA	Cell Signaling #2144	1:2000 in 4% skim milk
UCP1	GeneTex, GTX10983	1:1000 in 4% skim milk
anti-rabbit 2nd	Thermo Fisher #31460	1:2000 in 4% skim milk
anti-mouse 2nd	Thermo Fisher #31430	1:2000 in 4% skim milk

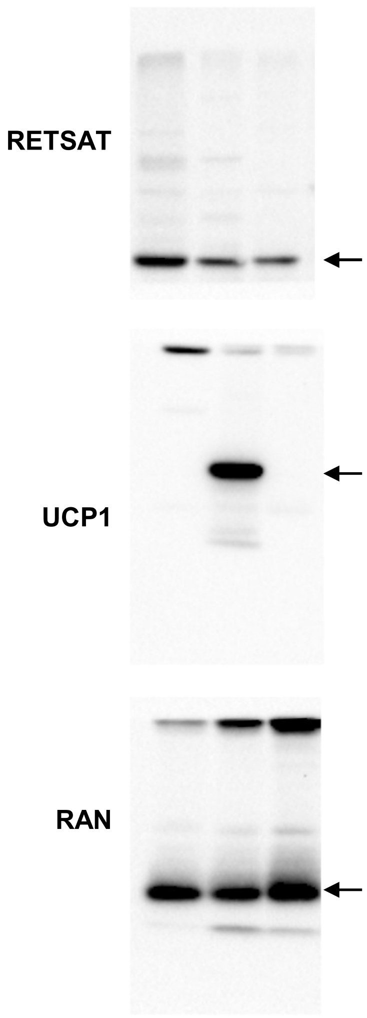

1A mouse tissues

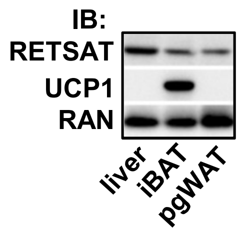

1D

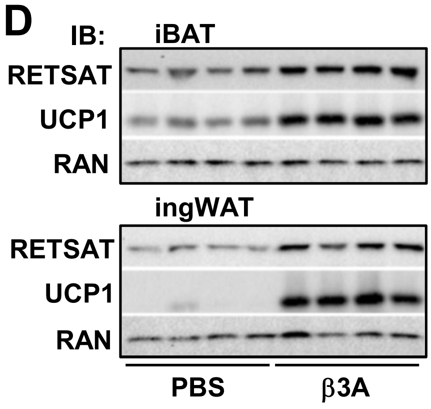
IB:
iBAT
RETSAT
UCP1
RAN
ingWAT
RETSAT
UCP1
RAN
PBS
β3A

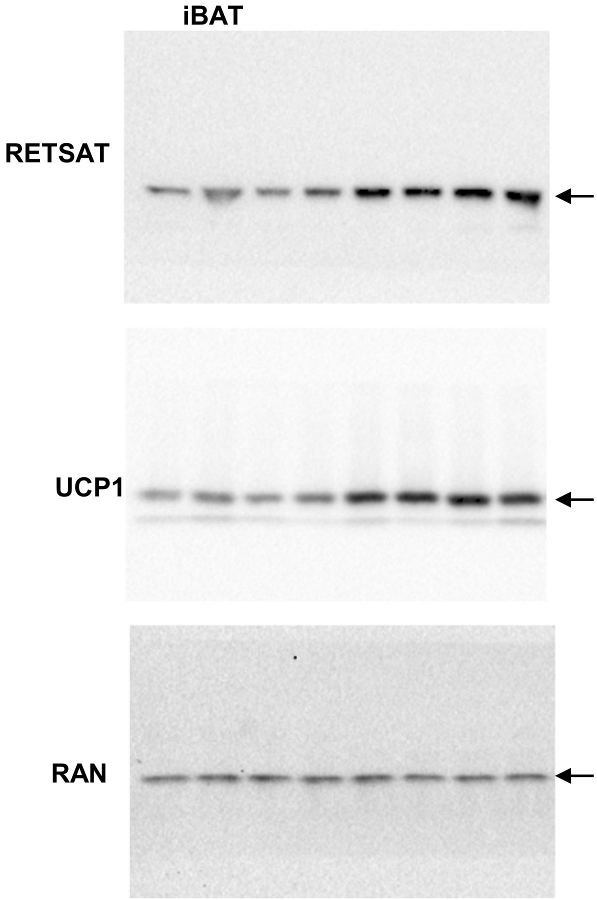
iBAT
RETSAT
UCP1
RAN

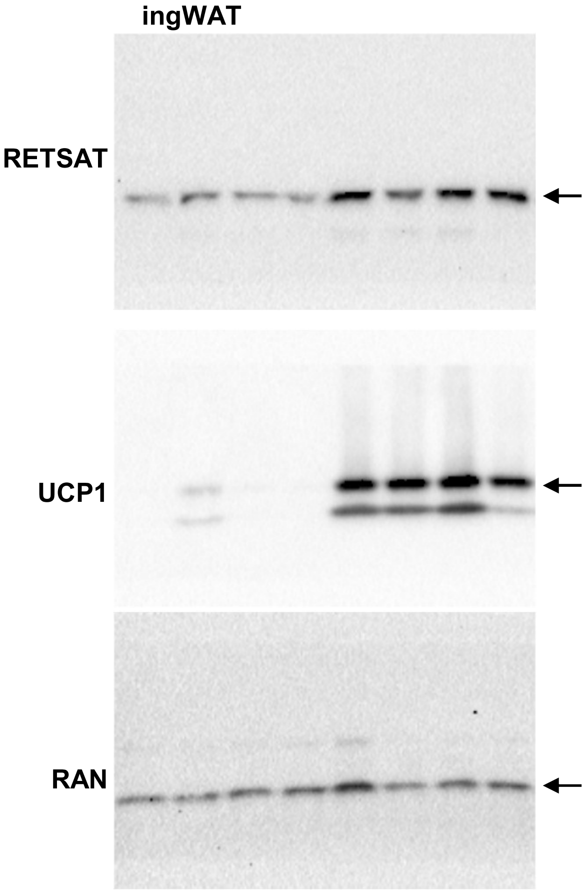
ingWAT
RETSAT
UCP1
RAN

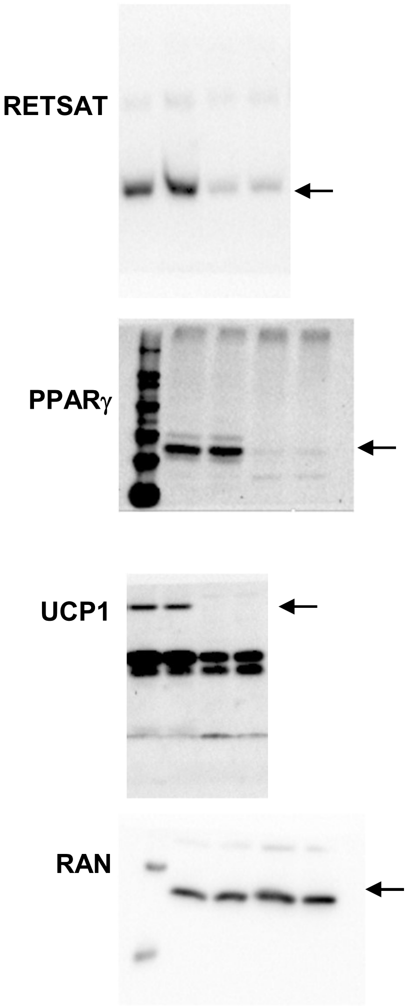

2A

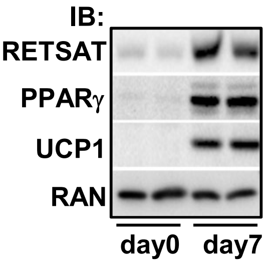

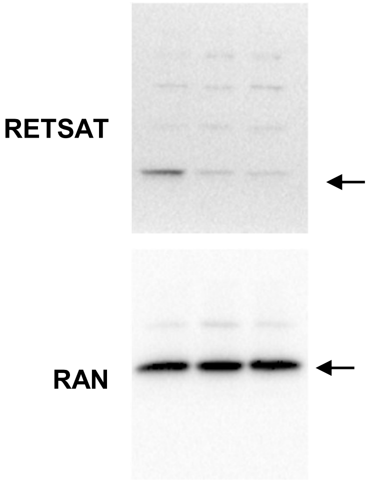
RETSAT
RAN

2C

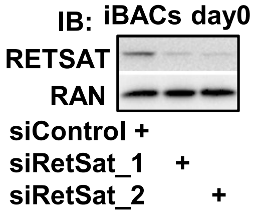
IB: iBACs day0
RETSAT
RAN
siControl +
siRetSat_1 +
siRetSat_2 +

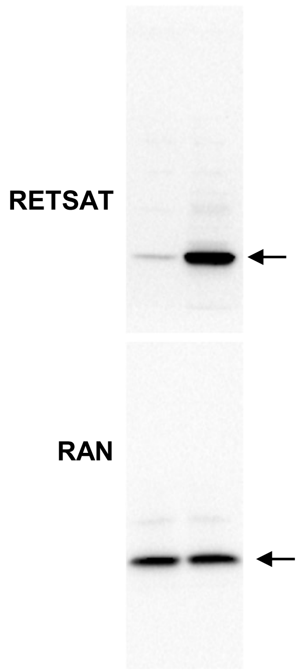

RETSAT
RAN

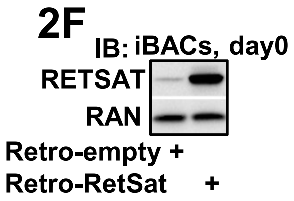

2F
IB: iBACs, day0
RETSAT
RAN
Retro-empty +
Retro-RetSat +

3C

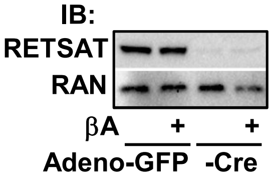

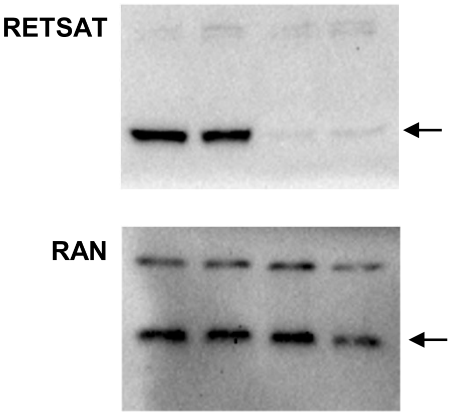

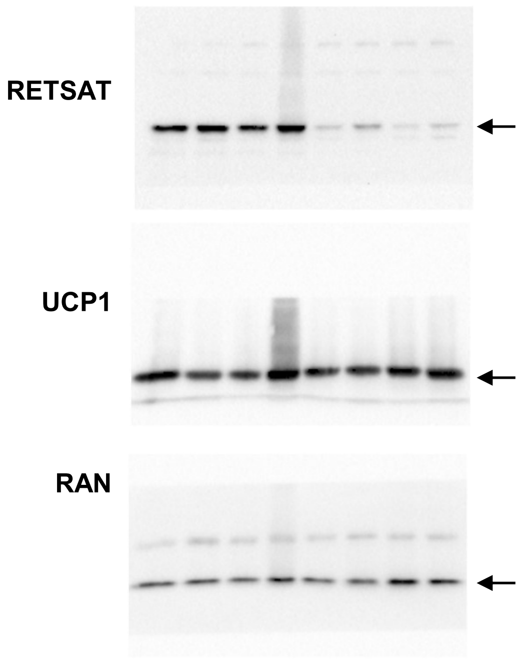

5A

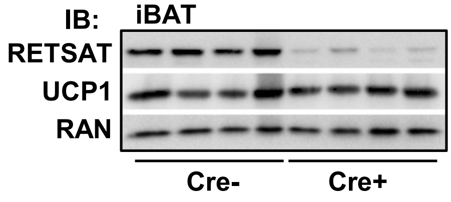

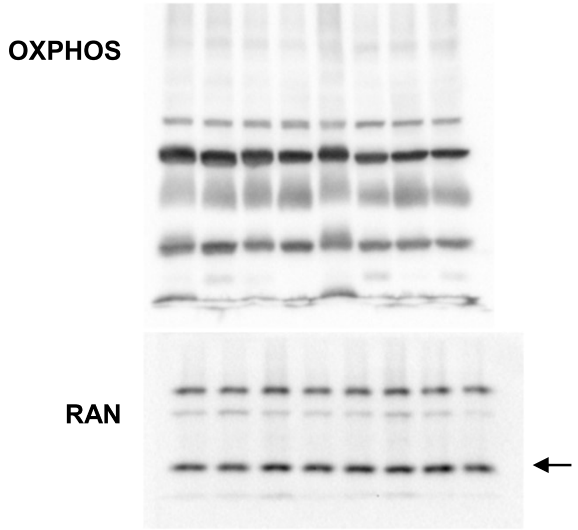
OXPHOS
RAN

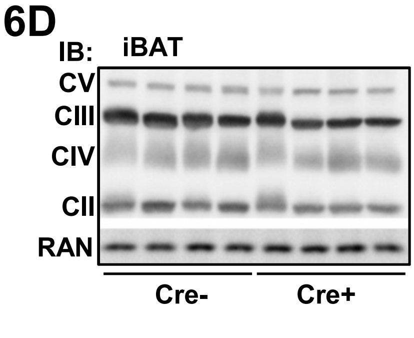
6D
IB:
iBAT
CV
CIII
CIV
CII
RAN
Cre-
Cre+

S3B

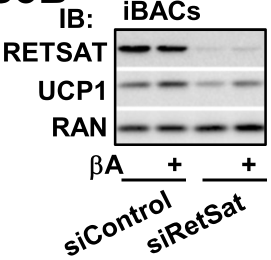
IB:
iBACs
RETSAT
UCP1
RAN
βA
+
+
siControl
siRetSat

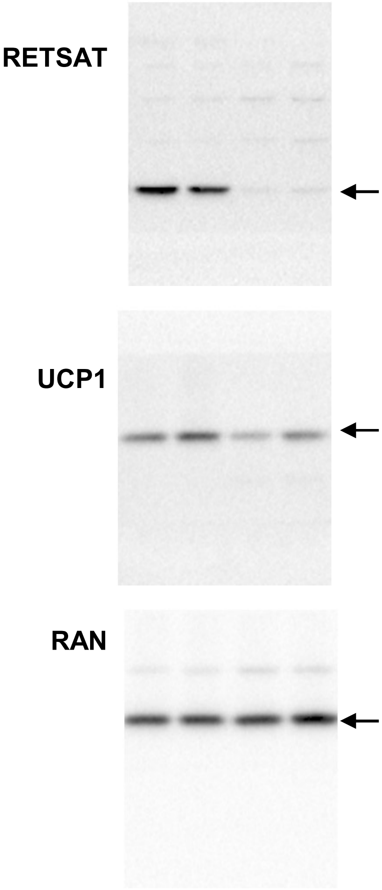
RETSAT
UCP1
RAN

S3D

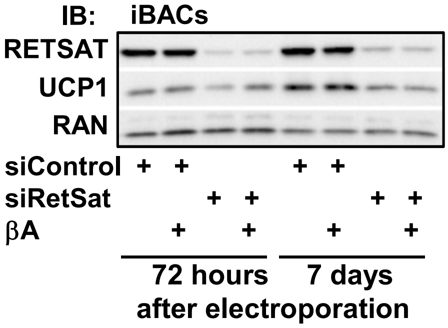

RETSAT

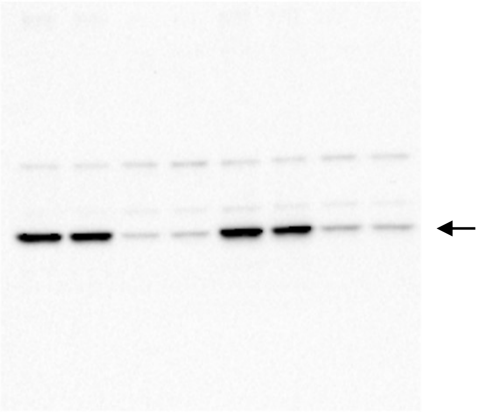

UCP1

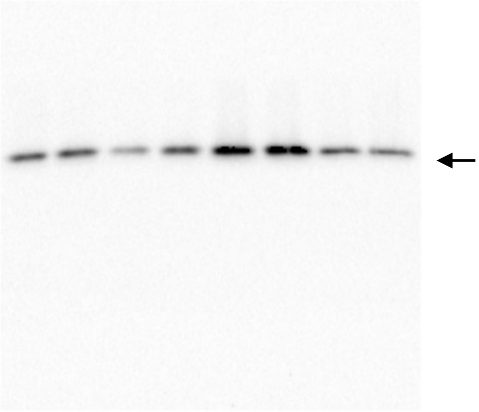

RAN

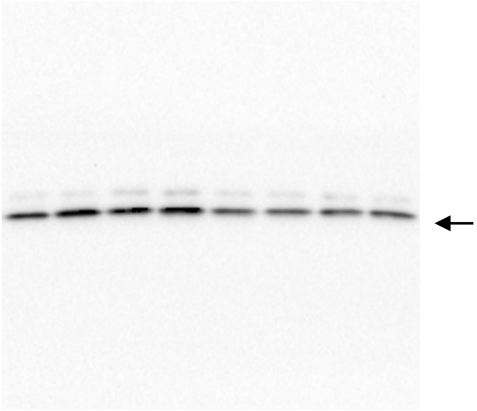

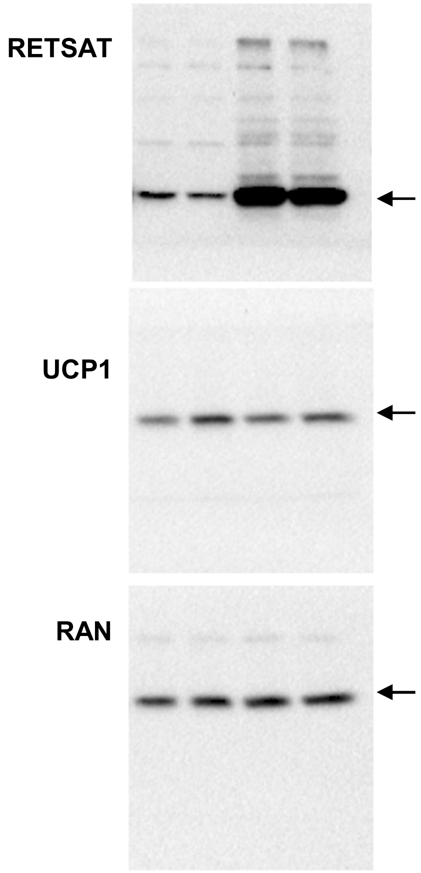
RETSAT
UCP1
RAN

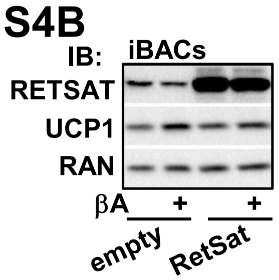
S4B
IB:
iBACs
RETSAT
UCP1
RAN
βA
+
+
empty
RetSat

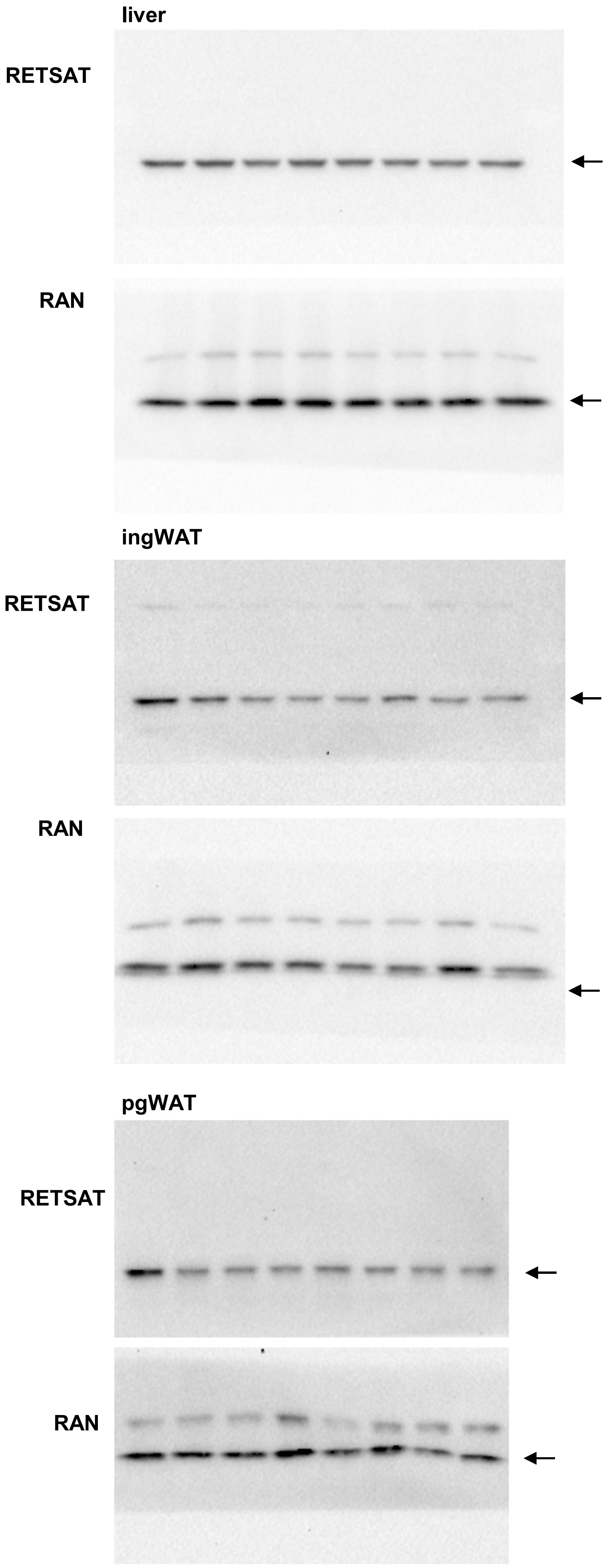
liver
RETSAT
RAN
ingWAT
RETSAT
RAN
pgWAT
RETSAT
RAN

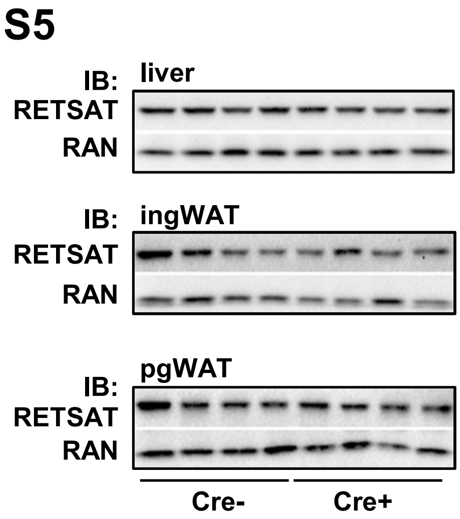
S5
IB: liver
RETSAT
RAN
IB: ingWAT
RETSAT
RAN
IB: pgWAT
RETSAT
RAN
Cre-
Cre+

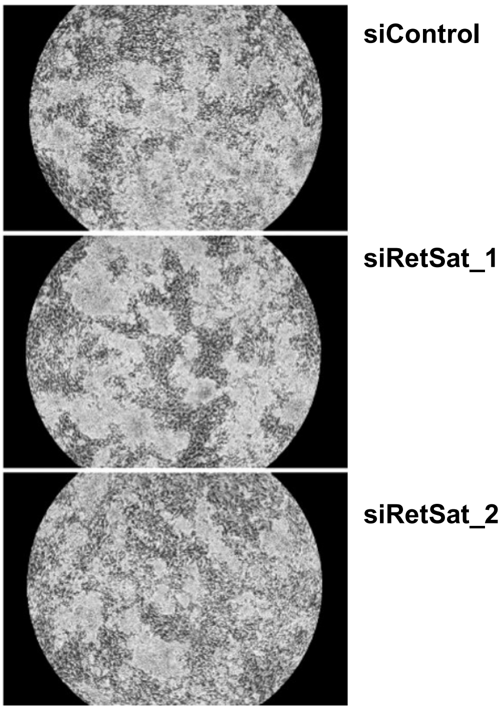
siControl
siRetSat_1
siRetSat_2

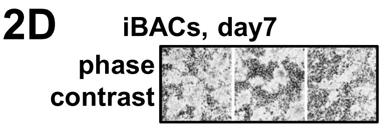
2D
iBACs, day7
phase
contrast

weak induction

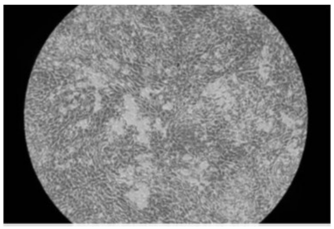

Retro-empty

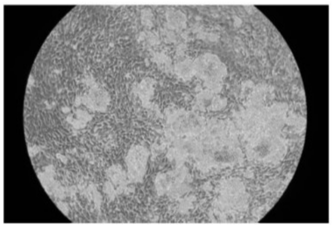

Retro-RetSat

strong induction

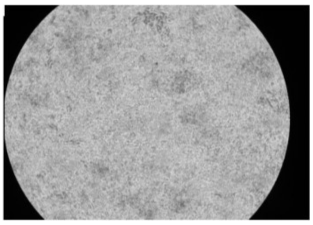

Retro-empty

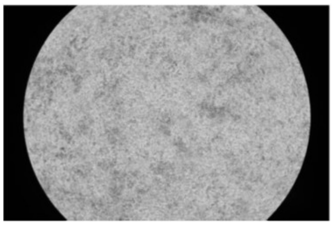

Retro-RetSat

2G

iBACs, day7

phase contrast

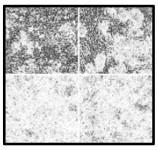

- pio

+ pio

Retro-empty +

Retro-RetSat +

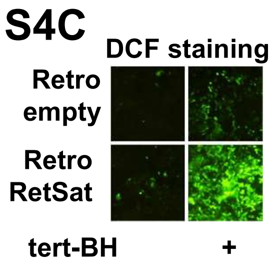
S4C
DCF staining
Retro
empty
Retro
RetSat
tert-BH
+

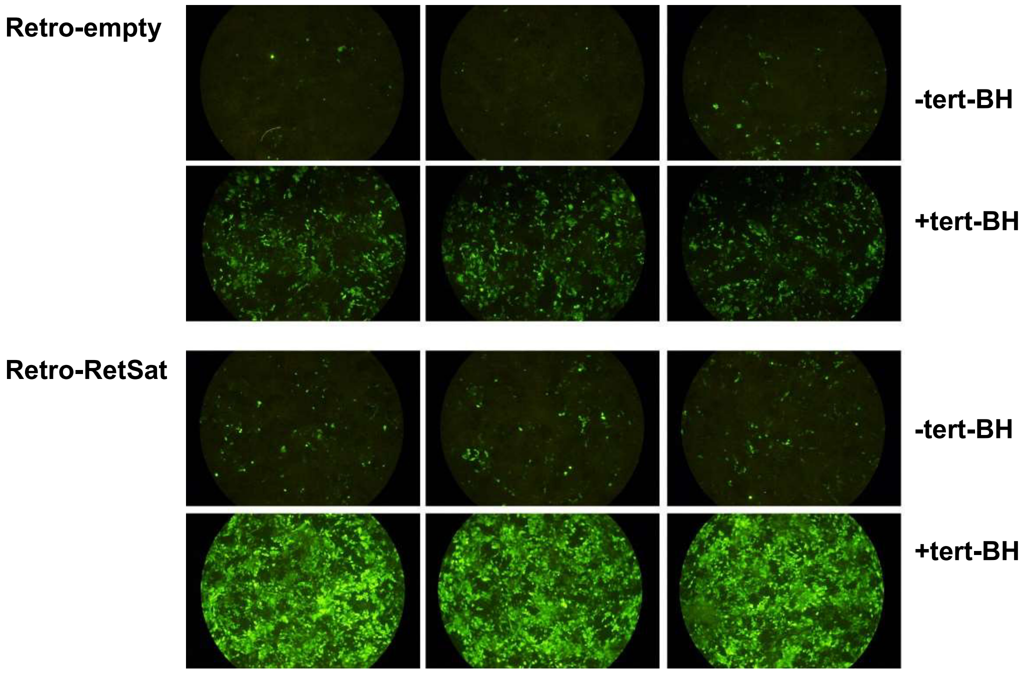
Retro-empty
-tert-BH
+tert-BH
Retro-RetSat
-tert-BH
+tert-BH
